# Protein profiling of human lung telocytes and microvascular endothelial cells using iTRAQ quantitative proteomics

**DOI:** 10.1111/jcmm.12350

**Published:** 2014-07-24

**Authors:** Yonghua Zheng, Dragos Cretoiu, Guoquan Yan, Sanda Maria Cretoiu, Laurentiu M Popescu, Hao Fang, Xiangdong Wang

**Affiliations:** a Fudan University Center for Clinical Bioinformatics, Zhongshan Hospital, Fudan University School of MedicineShanghai, China; b Division of Cellular and Molecular Medicine, Carol Davila University of Medicine and PharmacyBucharest, Romania; c Department of Molecular Medicine, Victor Babeş National Institute of PathologyBucharest, Romania; d Department of Chemistry, Institute of Biomedical Sciences, Fudan UniversityShanghai, China; e Department of Ultrastructural Pathology, Victor Babeş National Institute of PathologyBucharest, Romania; f Division of Advanced Studies, Victor Babes National Institute of PathologyBucharest, Romania; g Department of Anesthesiology, Zhongshan HospitalShanghai, China; h Jinshan Hospital Fudan UniversityShanghai, China

**Keywords:** telocytes, microvascular endothelial cells, proteomics, iTRAQ, LC-MS/MS, lung, intercellular signalling

## Abstract

Telocytes (TCs) are described as a particular type of cells of the interstitial space (www.telocytes.com). Their main characteristics are the very long telopodes with alternating podoms and podomers. Recently, we performed a comparative proteomic analysis of human lung TCs with fibroblasts, demonstrating that TCs are clearly a distinct cell type. Therefore, the present study aims to reinforce this idea by comparing lung TCs with endothelial cells (ECs), since TCs and ECs share immunopositivity for CD34. We applied isobaric tag for relative and absolute quantification (iTRAQ) combined with automated 2-D nano-ESI LC-MS/MS to analyse proteins extracted from TCs and ECs in primary cell cultures. In total, 1609 proteins were identified in cell cultures. 98 proteins (the 5th day), and 82 proteins (10th day) were confidently quantified (screened by two-sample *t*-test, *P* < 0.05) as up- or down-regulated (fold change >2). We found that in TCs there are 38 up-regulated proteins at the 5th day and 26 up-regulated proteins at the 10th day. Bioinformatics analysis using Panther revealed that the 38 proteins associated with TCs represented cellular functions such as intercellular communication (*via* vesicle mediated transport) and structure morphogenesis, being mainly cytoskeletal proteins and oxidoreductases. In addition, we found 60 up-regulated proteins in ECs *e.g*.: cell surface glycoprotein MUC18 (15.54-fold) and von Willebrand factor (5.74-fold). The 26 up-regulated proteins in TCs at 10th day, were also analysed and confirmed the same major cellular functions, while the 56 down-regulated proteins confirmed again their specificity for ECs. In conclusion, we report here the first extensive comparison of proteins from TCs and ECs using a quantitative proteomics approach. Our data show that TCs are completely different from ECs. Protein expression profile showed that TCs play specific roles in intercellular communication and intercellular signalling. Moreover, they might inhibit the oxidative stress and cellular ageing and may have pro-proliferative effects through the inhibition of apoptosis. The group of proteins identified in this study needs to be explored further for the role in pathogenesis of lung disease.

## Introduction

Telocytes (TCs) were identified as a new cell type of the stromal space [1] (details at www.telocytes.com). TCs were described in the trachea and lungs [2–5], besides other important locations: heart [6–9], female reproductive system [10–13], skin [14,15], digestive system [16–19], liver [20], urinary tract [21,22], prostate [23,24], skeletal muscle and neuromuscular spindles [25,26], eye [27], *etc*. Ultrastructurally, TCs are defined and easily to distinguish by their extremely long telopodes (tens up to hundreds of micrometres), an alternation of podoms and podomeres.

Recently, electrophysiological properties were described for TCs [28–30], their microarray-based gene expression analysis and microRNA signature were established [31,32] and some of their genomic features were revealed [33]. In a previous study, we reported the proteomic profile differences between TCs and fibroblasts [34].

Since their description, it became clear that TCs develop a 3D network through the organ interstitial space and are frequently detected in close relationships with organ-specific structures, blood capillaries, nerve endings and even with stem cells niches and immune cells [11,16,29]. Numerous studies have described the unusual immunophenotype of the TCs providing a list of molecular markers such as CD34, PDGFR α and β, CD117 [20,25,35–37]. Some of these markers are also expressed on endothelial cells (low level of CD34) and on pericytes (PDGFR α and β). However ECs co-express CD31 and pericytes co-express α-SMA, while TCs do not [25]. These similarities might be suggestive for a common mesodermal pre-cursor for TCs, ECs and for a perivascular origin of mesenchymal stem cells (for reviews see 38–42).

It is largely accepted that ECs in culture are subjected to phenotypic drift because of the lack of *in vivo* typical conditions [43], mainly oxygen exposure which is higher *in vitro*. These aspects together with the fact that proteomic studies also point out the differences between venous and arterial ECs [44] should lead us to the idea that *in vitro* culture studies should be viewed with circumspection without out-looking *in vivo* physiological influences. A study by Nguyen *et al*., regarding differential proteomic analysis of lymphatic, venous and arterial endothelial cells extracted from bovine mesenteric vessels underline the lack of substantial overlap between results from different research groups [45].

The present study shows the proteomic analysis of the TCs, by comparing them with ECs using iTRAQ labelling to identify the differentially expressed proteins. We think that the identification of a panel of 98 proteins at 5th day, and 82 proteins at 10th day in cell cultures, may represent the most differentially expressed proteins between TCs and ECs. We found that 38 proteins were overexpressed in TCs compared to ECs (at 5th day) and that 26 proteins were overexpressed in TCs compared to ECs (at 10th day). Bioinformatics analysis of the up-regulated proteins came again to confirm the involvement of TCs in intercellular communication, oxidative stress and cellular ageing. Also, TCs may have pro-proliferative effects through the inhibition of apoptosis.

## Material and methods

### Cell lines and tissue sampling

Human lung samples were obtained from the patients undergoing surgery for lung cancer. Lung fragments were removed from normal tissue area located at least at 15 cm from the tumour tissue. All tissue samples were obtained in accordance with a protocol approved by the Ethical Evaluation Committee of Zhongshan Hospital, Fudan University, Shanghai, China. Samples were processed within 30 min. from surgery. Cells were cultured using the protocol previously described [34]. Human pulmonary microvascular endothelial cell line was obtained from ScienCell Research Laboratories (Cat. no. 3000; Carlsbad, CA, USA).

### Cell culture and lysis

Cells from primary culture were used for the experiments. Cells (1 × 10^5^) were placed in 10-cm dishes with 10 ml high glucose DMEM (Gibco, Grand Island, NY, USA) complete medium, including 10% foetal calf serum (Gibco, Grand Island, NY, USA), 100 UI/ml penicillin and 0.1 mg/ml streptomycin (Sigma Chemical, St. Louis, MO, USA) in a humidified atmosphere of 5% CO_2_ at 370°C. Confluent cells were trypsinized at day 5 and day 10 respectively. Approximately 10^6^ cells from day 5 or day 10 were re-suspended in a solution of 9.5 moles/l urea, 1% dithiothreitol, 40 mg/ml protease inhibitor cocktail, 0.2 mmoles/l Na_2_VO_3_ and 1 mmole/l NaF. The mixture was incubated and stirred by end-over-end rotation at 4°C for 60 min. The resultant suspension was centrifuged at 40,000 × g for 1 hr at 15°C. The supernatant was stored in small aliquots at −80°C, and the protein concentration was determined using a modified Bradford method.

### Automated 2-D nano-ESI LC-MS/MS analysis of peptides

Proteins extracted from primary cultures of TCs and EC were analysed by automated 2-dimensional nano-electrospray ionization liquid chromatography tandem mass spectrometry as was previously described [46,47].

### Sample preparation

The samples were ground in liquid nitrogen. One millilitre of lysis buffer (8 M urea, 1× Protease Inhibitor Cocktail (Roche Ltd. Basel, Switzerland)) was added to sample, followed by sonication on ice and centrifugation at 29,000 × g for 10 min. at 4°C. The supernatant was transferred to a fresh tube, and stored at −80°C until needed.

### iTRAQ labelling and protein digestion

For each sample, proteins were precipitated with ice-cold acetone, and then were redissolved in the dissolution buffer (0.5 M triethylammonium bicarbonate, 0.1% SDS). Then proteins were quantified by BCA protein assay, and 100 μg of protein was tryptically digested and the resultant peptide mixture was labelled using chemicals from the iTRAQ reagent kit (Applied Biosystems, Foster City, CA, USA). Disulphide bonds were reduced in 5 mM Tris-(2-carboxyethy) phosphine for 1 hr at 60°C, followed by blocking cysteine residues in 10 mM methyl methanethiosulfonate for 30 min. at room temperature, before digestion with sequence-grade modified trypsin (Promega, Madison, WI, USA). For labelling, each iTRAQ reagent was dissolved in 50 μl of isopropanol and added to the respective peptide mixture.

Proteins were labelled with the iTRAQ tags as follows: Pulmonary microvascular endothelial cells (5 days) - 113 isobaric tag, TCs (5 days) - 116 isobaric tag, Pulmonary microvascular endothelial cells (10 days) - 117 isobaric tag, TCs (10 days) - 121 isobaric tag. The labelled samples were combined and dried in vacuo. A SepPac™ C18 cartridge (1 cm^3^/50 mg; Waters Corporation, Milford, MA, USA) was used to remove the salt buffer and then was dried in a vacuum concentrator for the next step.

### High pH reverse phase separation

Using a described protocol [48], the peptide mixture was redissolved in the buffer A (buffer A: 20 mM ammonium formate in water, pH 10.0, adjusted with ammonium hydroxide), and then fractionated by high pH separation using a Aquity UPLC system (Waters Corporation) connected to a reverse phase column (XBridge C18 column, 2.1 mm × 150 mm, 3.5 μm, 300 Å; Waters Corporation). High pH separation was performed with a linear gradient. Starting from 2% B to 40% B in 45 min. (B: 20 mM ammonium formate in 90% ACN, pH 10.0, adjusted with ammonium hydroxide). The column was re-equilibrated at initial conditions for 15 min. The column flow rate was maintained at 200 μl/min. and column temperature was maintained at room temperature. Fourteen fractions were collected, and each fraction was dried in a vacuum concentrator for the next step.

### Low pH nano-HPLC-MS/MS analysis

The peptides were re-suspended with 80 μl solvent C (C: water with 0.1% formic acid; D: ACN with 0.1% formic acid), separated by nanoLC and analysed by on-line electrospray tandem mass spectrometry. The experiments were performed on a Nano Aquity UPLC system (Waters Corporation) connected to an LTQ Orbitrap XL mass spectrometer (Thermo Electron Corp., Bremen, Germany) equipped with an online nanoelectrospray ion source (Michrom Bioresources, Auburn, CA, USA). 20 μl peptide sample was loaded onto the Thermo Scientific Acclaim PepMap C18 column (100 μm × 2 cm, 3 μm particle size), with a flow of 10 μl/min. for 5 min. and subsequently separated on the analytical column (Acclaim PepMap C18, 75 μm × 15 cm) with a linear gradient, from 5% D to 45% D in 165 min. The column was re-equilibrated at initial conditions for 15 min. The column flow rate was maintained at 300 nl/min. and column temperature was maintained at 35°C. The electrospray voltage of 1.9 kV *versus* the inlet of the mass spectrometer was used.

LTQ Orbitrap XL mass spectrometer was operated after a protocol previously described [34].

### Database searching and criteria

Protein identification and quantification for the iTRAQ experiment was performed with the ProteinPilot software version 4.0 (Applied Biosystems). The database was the Human UniProtKB/Swiss-Prot database (Release 2011_10_15, with 20248 sequences). The Paragon Algorithm in ProteinPilot software was used for peptide identification and isoform specific quantification. The detailed method of ProteinPilot analysis was described previously [34]. For iTRAQ quantification, the peptide for quantification was automatically selected by Pro Group algorithm (at least two peptides with 99% confidence) to calculate the reporter peak area, error factor and *P*-value. For the selection of differentially expressed proteins, we considered the following situation: (1) the proteins must contain at least two unique high-scoring peptides; (2) the proteins must have a *P* < 0.05, and the proteins identified with mass tag changes ratio must be ≥1.3 or ≤0.75. Differentially expressed proteins were screened by two-sample t-test (*P* < 0.05) and fold change (>2), based on the bioinformatics analysis.

The biological interpretation of the results was aided by PANTHER (Protein ANalysis THrough Evolutionary Relationships) Classification System annotations (http://www.pantherdb.org/).

Heat maps were created after MS/MS fragmentation spectra were analysed using PEAKS search engine tool (PEAKS Studio 7; Bioinformatics Solutions Inc., Waterloo, ON, Canada).

We also used the Search Tool for the Retrieval of Interacting Genes/Proteins (STRING) (http://www.string-db.org/) database of physical and functional interactions to evaluate the interactions among the up-regulated proteins of the TCs and ECs. Bonferroni correction was used as a conservative method to control the family wise error rate when highlighting proteins involved in different biological processes.

## Results

Quantitative proteomics has been used to evaluate the differentially expressed proteins in TCs and ECs. We compared the protein expression profiles between those two cell types, at different moments in time (5th day and 10th day in cell culture). In particular, we identified a total of 1609 proteins of which 98 satisfied our filtering criteria of proteins that exhibited fold changes ≥2 in TCs *versus* ECs at day 5 (Table1), and 82 proteins in TCs *versus* ECs at day 10 (Table2), respectively.

**Table 1:** Selected list of top 98 proteins identified with more than twofold change in TCs *versus* ECs at 5th day sorted by iTRAQ ratio and presenting the number of peptides hits

Accession	Protein name	Peptides (95%)	%Cov (95)	iTRAQ ratio ECs:TCs	Fold enrichment in TCs	*P*
MYH14_HUMAN	Myosin-14	18	7.67	0.053	18.84	0.0002
SODM_HUMAN	Superoxide dismutase [Mn], mitochondrial	10	35.14	0.069	14.59	0.0002
ASAH1_HUMAN	Acid ceramidase	2	5.82	0.131	7.63	0.0315
EVPL_HUMAN	Envoplakin	1	0.79	0.161	6.22	0.0346
EPIPL_HUMAN	Epiplakin	17	16.07	0.167	5.99	0.0250
HBB_HUMAN	Haemoglobin subunit beta	2	15.65	0.219	4.56	0.0466
HBA_HUMAN	Haemoglobin subunit alpha	2	16.90	0.244	4.09	0.0055
THIM_HUMAN	3-ketoacyl-CoA thiolase, mitochondrial	12	33.50	0.245	4.08	0.0186
SQRD_HUMAN	Sulphide:quinone oxidoreductase, mitochondrial	9	19.78	0.245	4.08	0.0015
LMO7_HUMAN	LIM domain only protein 7	3	2.02	0.246	4.06	0.0246
FRIL_HUMAN	Ferritin light chain	1	8.57	0.252	3.97	0.0178
ALDH2_HUMAN	Aldehyde dehydrogenase, mitochondrial	5	10.06	0.253	3.96	0.0152
TAGL_HUMAN	Transgelin	15	75.12	0.258	3.88	0.0058
ACADV_HUMAN	Very long-chain specific acyl-CoA dehydrogenase, mitochondrial	6	9.62	0.301	3.33	0.0325
TPSN_HUMAN	Tapasin	3	5.13	0.318	3.14	0.0292
PGRC1_HUMAN	Membrane-associated progesterone receptor component 1	4	18.97	0.328	3.04	0.0068
SUCA_HUMAN	Succinyl-CoA ligase [GDP-forming] subunit alpha, mitochondrial	5	17.63	0.344	2.90	0.0018
ICAM1_HUMAN	Intercellular adhesion molecule 1	6	14.66	0.374	2.67	0.0021
TFR1_HUMAN	Transferrin receptor protein 1	5	5.79	0.378	2.65	0.0073
NLTP_HUMAN	Non-specific lipid-transfer protein	9	12.43	0.390	2.57	0.0013
CO1A1_HUMAN	Collagen alpha-1(I) chain	4	3.01	0.407	2.46	0.0290
AT1A1_HUMAN	Sodium/potassium-transporting ATPase subunit alpha-1	17	18.77	0.412	2.43	0.0000
COX5B_HUMAN	Cytochrome c oxidase subunit 5B, mitochondrial	4	34.11	0.438	2.28	0.0042
DHB4_HUMAN	Peroxisomal multifunctional enzyme type 2	11	25.00	0.442	2.26	0.0000
MYH10_HUMAN	Myosin-10	45	19.59	0.445	2.25	0.0240
KAD3_HUMAN	GTP:AMP phosphotransferase, mitochondrial	2	10.57	0.451	2.22	0.0107
CO4A_HUMAN	Complement C4-A	4	2.52	0.460	2.17	0.0215
COX5A_HUMAN	Cytochrome c oxidase subunit 5A, mitochondrial	6	58.67	0.470	2.13	0.0015
DLDH_HUMAN	Dihydrolipoyl dehydrogenase, mitochondrial	10	27.50	0.472	2.12	0.0047
ALBU_HUMAN	Serum albumin	11	14.45	0.473	2.11	0.0098
A2MG_HUMAN	Alpha-2-macroglobulin	7	4.34	0.476	2.10	0.0268
ETHE1_HUMAN	Protein ETHE1, mitochondrial	2	9.45	0.480	2.08	0.0370
KAD2_HUMAN	Adenylate kinase 2, mitochondrial	8	41.42	0.487	2.05	0.0004
ERGI1_HUMAN	Endoplasmic reticulum-Golgi intermediate compartment protein 1	4	18.97	0.488	2.05	0.0258
ERP29_HUMAN	Endoplasmic reticulum resident protein 29	7	28.74	0.490	2.04	0.0015
GRP75_HUMAN	Stress-70 protein, mitochondrial	32	40.94	0.493	2.03	0.0114
ETFA_HUMAN	Electron transfer flavoprotein subunit alpha, mitochondrial	10	38.44	0.494	2.02	0.0002
CH60_HUMAN	60 kD heat shock protein, mitochondrial	60	62.30	0.496	2.02	0.0011

Accession	Protein name	Peptides (95%)	%Cov (95)	iTRAQ ratio ECs:TCs	Fold enrichment in ECs	*P*
PUR6_HUMAN	Multifunctional protein ADE2	4	6.59	2.007	2.01	0.0375
G6PI_HUMAN	Glucose-6-phosphate isomerase	4	7.35	2.019	2.02	0.0029
RS13_HUMAN	40S ribosomal protein S13	5	29.80	2.025	2.02	0.0035
PROF1_HUMAN	Profilin-1	23	75.71	2.026	2.03	0.0004
TXND5_HUMAN	Thioredoxin domain-containing protein 5	15	40.51	2.030	2.03	0.0011
LEG1_HUMAN	Galectin-1	27	91.11	2.053	2.05	0.0001
ARC1B_HUMAN	Actin-related protein 2/3 complex subunit 1B	2	5.11	2.056	2.06	0.0126
AMPN_HUMAN	Aminopeptidase N	7	7.03	2.061	2.06	0.0162
LDHB_HUMAN	L-lactate dehydrogenase B chain	8	23.35	2.094	2.09	0.0021
ACLY_HUMAN	ATP-citrate synthase	11	11.08	2.096	2.10	0.0004
THIO_HUMAN	Thioredoxin	5	51.43	2.115	2.11	0.0022
GDIB_HUMAN	Rab GDP-dissociation inhibitor beta	5	14.83	2.118	2.12	0.0007
EEA1_HUMAN	Early endosome antigen 1	4	2.62	2.140	2.14	0.0111
TGM2_HUMAN	Protein-glutamine gamma-glutamyltransferase 2	18	21.25	2.145	2.15	0.0351
RL19_HUMAN	60S ribosomal protein L19	3	14.80	2.207	2.21	0.0133
COF1_HUMAN	Cofilin-1	21	71.69	2.210	2.21	0.0138
FLNB_HUMAN	Filamin-B	96	41.78	2.210	2.21	0.0000
H15_HUMAN	Histone H1.5	7	23.45	2.219	2.22	0.0186
RAN_HUMAN	GTP-binding nuclear protein Ran	7	32.87	2.245	2.25	0.0117
NQO1_HUMAN NAD(P)	H dehydrogenase [quinone] 1	2	7.66	2.246	2.25	0.0206
HS90B_HUMAN	Heat shock protein HSP 90-beta	37	35.77	2.275	2.27	0.0006
RCN1_HUMAN	Reticulocalbin-1	8	22.66	2.283	2.28	0.0093
CDC37_HUMAN	Hsp90 co-chaperone Cdc37	10	27.25	2.306	2.31	0.0005
G3P_HUMAN	Glyceraldehyde-3-phosphate dehydrogenase	42	64.48	2.338	2.34	0.0045
SH3L3_HUMAN	SH3 domain-binding glutamic acid-rich-like protein 3	3	34.41	2.341	2.34	0.0160
ITA5_HUMAN	Integrin alpha-5	7	8.20	2.366	2.37	0.0011
EHD2_HUMAN	EH domain-containing protein 2	8	17.13	2.374	2.37	0.0009
PPIA_HUMAN	Peptidyl-prolyl cis-trans isomerase A	17	75.76	2.383	2.38	0.0001
MOES_HUMAN	Moesin	26	44.71	2.394	2.39	0.0054
PRDX1_HUMAN	Peroxiredoxin-1	14	46.73	2.419	2.42	0.0006
UCHL1_HUMAN	Ubiquitin carboxyl-terminal hydrolase isozyme L1	7	37.22	2.486	2.49	0.0033
TAGL2_HUMAN	Transgelin-2	19	74.87	2.533	2.53	0.0000
HPRT_HUMAN	Hypoxanthine-guanine phosphoribosyltransferase	2	11.01	2.570	2.57	0.0220
PLST_HUMAN	Plastin-3	11	19.37	2.570	2.57	0.0000
WDR1_HUMAN	WD repeat-containing protein 1	10	18.15	2.577	2.58	0.0000
UB2L3_HUMAN	Ubiquitin-conjugating enzyme E2 L3	1	4.54	2.601	2.60	0.0052
HMGB1_HUMAN	High mobility group protein B1	3	15.35	2.614	2.61	0.0014
TRXR1_HUMAN	Thioredoxin reductase 1, cytoplasmic	9	16.49	2.618	2.62	0.0001
NSF1C_HUMAN	NSFL1 cofactor p47	4	19.19	2.696	2.70	0.0192
APEX1_HUMAN	DNA-(apurinic or apyrimidinic site) lyase	2	6.60	2.718	2.72	0.0292
K6PP_HUMAN	6-phosphofructokinase type C	5	8.29	2.741	2.74	0.0004
HINT1_HUMAN	Histidine triad nucleotide-binding protein 1	3	40.48	2.775	2.77	0.0244
STIP1_HUMAN	Stress-induced-phosphoprotein 1	11	19.34	2.777	2.78	0.0000
RL15_HUMAN	60S ribosomal protein L15	2	7.84	2.792	2.79	0.0232
S10AD_HUMAN	Protein S100-A13	5	44.90	2.832	2.83	0.0016
CSRP1_HUMAN	Cysteine and glycine-rich protein 1	3	21.76	2.843	2.84	0.0280
VAT1_HUMAN	Synaptic vesicle membrane protein VAT-1 homologue	12	31.81	2.911	2.91	0.0012
6PGD_HUMAN	6-phosphogluconate dehydrogenase, decarboxylating	5	10.77	2.952	2.95	0.0346
SDPR_HUMAN	Serum deprivation-response protein	3	9.18	3.146	3.15	0.0278
GDIR1_HUMAN	Rho GDP-dissociation inhibitor 1	4	18.14	3.740	3.74	0.0010
FSCN1_HUMAN	Fascin	6	16.63	4.026	4.03	0.0012
HMGA1_HUMAN	High mobility group protein HMG-I/HMG-Y	2	22.43	4.281	4.28	0.0031
PECA1_HUMAN	Platelet endothelial cell adhesion molecule	9	11.52	4.492	4.49	0.0131
SCRN1_HUMAN	Secernin-1	2	5.80	4.937	4.94	0.0203
VWF_HUMAN	von Willebrand factor	19	7.93	5.740	5.74	0.0002
NEST_HUMAN	Nestin	22	16.84	5.924	5.92	0.0000
FKB1A_HUMAN	Peptidyl-prolyl cis-trans isomerase FKBP1A	4	34.26	6.650	6.65	0.0168
KCD12_HUMAN	BTB/POZ domain-containing protein KCTD12	3	9.85	7.264	7.26	0.0038
RAIN_HUMAN	Ras-interacting protein 1	1	1.14	13.416	13.42	0.0393
MUC18_HUMAN	Cell surface glycoprotein MUC18	7	12.69	15.540	15.54	0.0050

**Table 2:** Selected list of top 82 proteins identified with more than twofold change in TCs *versus* ECs at 10th day sorted by iTRAQ ratio and presenting the number of peptides hits

Accession	Protein name	Peptides (95%)	%Cov (95)	iTRAQ ratio ECs:TCs	Fold enrichment in TCs	*P*
PTGIS_HUMAN	Prostacyclin synthase	7	15.80	0.112	8.93	0.0016
MUC1_HUMAN	Mucin-1	2	1.75	0.199	5.02	0.0390
EPIPL_HUMAN	Epiplakin	17	16.07	0.209	4.78	0.0083
SODM_HUMAN	Superoxide dismutase [Mn], mitochondrial	10	35.14	0.222	4.50	0.0155
AL1B1_HUMAN	Aldehyde dehydrogenase X, mitochondrial	5	13.93	0.246	4.06	0.0133
SERA_HUMAN	D-3-phosphoglycerate dehydrogenase	4	8.07	0.295	3.39	0.0019
CYB5_HUMAN	Cytochrome b5	4	32.09	0.306	3.27	0.0483
SQRD_HUMAN	Sulphide:quinone oxidoreductase, mitochondrial	9	19.78	0.328	3.05	0.0000
THIM_HUMAN	3-ketoacyl-CoA thiolase, mitochondrial	12	33.50	0.338	2.95	0.0000
ERGI1_HUMAN	Endoplasmic reticulum-Golgi intermediate compartment protein 1	4	18.97	0.348	2.88	0.0088
EZRI_HUMAN	Ezrin	19	31.40	0.354	2.83	0.0162
CP51A_HUMAN	Lanosterol 14-alpha demethylase	2	3.98	0.361	2.77	0.0156
PLOD2_HUMAN	Procollagen-lysine,2-oxoglutarate 5-dioxygenase 2	15	22.39	0.370	2.70	0.0061
CO1A2_HUMAN	Collagen alpha-2(I) chain	9	7.91	0.382	2.62	0.0001
ECH1_HUMAN	Delta(3,5)-Delta(2,4)-dienoyl-CoA isomerase, mitochondrial	4	23.78	0.385	2.60	0.0099
RRS1_HUMAN	Ribosome biogenesis regulatory protein homologue	3	9.86	0.393	2.54	0.0059
ACADV_HUMAN	Very long-chain specific acyl-CoA dehydrogenase, mitochondrial	6	9.62	0.414	2.41	0.0449
NB5R1_HUMAN	NADH-cytochrome b5 reductase 1	1	2.95	0.414	2.41	0.0084
NOP2_HUMAN	Putative ribosomal RNA methyltransferase NOP2	3	4.06	0.436	2.29	0.0138
PGRC1_HUMAN	Membrane-associated progesterone receptor component 1	4	18.97	0.440	2.27	0.0210
FINC_HUMAN	Fibronectin	35	20.03	0.453	2.21	0.0002
DHB12_HUMAN	Estradiol 17-beta-dehydrogenase 12	3	11.54	0.454	2.20	0.0098
DHB4_HUMAN	Peroxisomal multifunctional enzyme type 2	11	25.00	0.479	2.09	0.0418
TAGL_HUMAN	Transgelin	15	75.12	0.482	2.08	0.0000
OAT_HUMAN	Ornithine aminotransferase, mitochondrial	5	14.12	0.487	2.05	0.0007
LPPRC_HUMAN	Leucine-rich PPR motif-containing protein, mitochondrial	12	9.83	0.497	2.01	0.0403

Accession	Protein name	Peptides (95%)	%Cov (95)	iTRAQ ratio ECs:TCs	Fold enrichment in ECs	*P*
H15_HUMAN	Histone H1.5	7	23.45	2.002	2.00	0.0037
RL7A_HUMAN	60S ribosomal protein L7a	6	21.80	2.009	2.01	0.0001
PLOD1_HUMAN	Procollagen-lysine,2-oxoglutarate 5-dioxygenase 1	13	20.22	2.063	2.06	0.0000
FETUA_HUMAN	Alpha-2-HS-glycoprotein	4	6.81	2.083	2.08	0.0373
ALDOA_HUMAN	Fructose-bisphosphate aldolase A	24	53.02	2.090	2.09	0.0000
PEA15_HUMAN	Astrocytic phosphoprotein PEA-15	2	16.92	2.100	2.10	0.0063
PROF1_HUMAN	Profilin-1	23	75.71	2.107	2.11	0.0005
TPIS_HUMAN	Triosephosphate isomerase	20	64.26	2.137	2.14	0.0000
CNN2_HUMAN	Calponin-2	9	37.86	2.156	2.16	0.0009
ENOA_HUMAN	Alpha-enolase	46	73.27	2.172	2.17	0.0000
DEST_HUMAN	Destrin	5	28.48	2.175	2.18	0.0118
SH3L3_HUMAN	SH3 domain-binding glutamic acid-rich-like protein 3	3	34.41	2.185	2.19	0.0196
H12_HUMAN	Histone H1.2	7	30.99	2.197	2.20	0.0056
TGM2_HUMAN	Protein-glutamine gamma-glutamyltransferase 2	18	21.25	2.205	2.21	0.0247
WDR1_HUMAN	WD repeat-containing protein 1	10	18.15	2.218	2.22	0.0000
GPX1_HUMAN	Glutathione peroxidase 1	6	23.15	2.259	2.26	0.0005
ACTN4_HUMAN	Alpha-actinin-4	44	42.15	2.269	2.27	0.0057
P3H3_HUMAN	Prolyl 3-hydroxylase 3	4	7.74	2.275	2.27	0.0138
COTL1_HUMAN	Coactosin-like protein	4	18.31	2.275	2.27	0.0125
ITA5_HUMAN	Integrin alpha-5	7	8.20	2.320	2.32	0.0001
RL24_HUMAN	60S ribosomal protein L24	6	31.21	2.329	2.33	0.0009
PDLI7_HUMAN	PDZ and LIM domain protein 7	4	8.97	2.329	2.33	0.0027
6PGD_HUMAN	6-phosphogluconate dehydrogenase, decarboxylating	5	10.77	2.360	2.36	0.0152
PLST_HUMAN	Plastin-3	11	19.37	2.388	2.39	0.0000
ZYX_HUMAN	Zyxin	10	18.53	2.515	2.51	0.0234
CSRP1_HUMAN	Cysteine and glycine-rich protein 1	3	21.76	2.525	2.53	0.0014
PDLI1_HUMAN	PDZ and LIM domain protein 1	6	26.75	2.529	2.53	0.0001
ACTN1_HUMAN	Alpha-actinin-1	37	38.68	2.557	2.56	0.0000
DPYL2_HUMAN	Dihydropyrimidinase-related protein 2	10	21.33	2.560	2.56	0.0032
NTF2_HUMAN	Nuclear transport factor 2	2	17.32	2.599	2.60	0.0162
MOES_HUMAN	Moesin	26	44.71	2.609	2.61	0.0004
SPHM_HUMAN	N-sulphoglucosamine sulphohydrolase	2	4.98	2.639	2.64	0.0049
EHD2_HUMAN	EH domain-containing protein 2	8	17.13	2.738	2.74	0.0007
AMPN_HUMAN	Aminopeptidase N	7	7.03	2.760	2.76	0.0290
VIME_HUMAN	Vimentin	200	83.69	2.833	2.83	0.0001
CATB_HUMAN	Cathepsin B	7	23.89	2.985	2.98	0.0041
PTRF_HUMAN	Polymerase I and transcript release factor	17	42.82	3.019	3.02	0.0019
FLNB_HUMAN	Filamin-B	96	41.78	3.050	3.05	0.0000
K6PP_HUMAN	6-phosphofructokinase type C	5	8.29	3.090	3.09	0.0001
TSP1_HUMAN	Thrombospondin-1	3	3.16	3.138	3.14	0.0056
VAT1_HUMAN	Synaptic vesicle membrane protein VAT-1 homologue	12	31.81	3.235	3.23	0.0000
HSPB1_HUMAN	Heat shock protein beta-1	17	61.46	3.306	3.31	0.0311
FSCN1_HUMAN	Fascin	6	16.63	3.473	3.47	0.0000
TPP1_HUMAN	Tripeptidyl-peptidase 1	7	12.08	3.573	3.57	0.0200
SDPR_HUMAN	Serum deprivation-response protein	3	9.18	3.951	3.95	0.0004
NEST_HUMAN	Nestin	22	16.84	4.134	4.13	0.0004
SYWC_HUMAN	Tryptophanyl-tRNA synthetase, cytoplasmic	3	8.70	4.389	4.39	0.0009
TXND5_HUMAN	Thioredoxin domain-containing protein 5	15	40.51	5.918	5.92	0.0000
EGLN_HUMAN	Endoglin	3	6.69	6.297	6.30	0.0067
FABP5_HUMAN	Fatty acid-binding protein, epidermal	4	16.30	7.134	7.13	0.0109
MUC18_HUMAN	Cell surface glycoprotein MUC18	7	12.69	9.313	9.31	0.0003
FKB1A_HUMAN	Peptidyl-prolyl cis-trans isomerase FKBP1A	4	34.26	10.535	10.53	0.0244
PECA1_HUMAN	Platelet endothelial cell adhesion molecule	9	11.52	13.313	13.31	0.0029
VWF_HUMAN	von Willebrand factor	19	7.93	15.888	15.89	0.0000
CRIP2_HUMAN	Cysteine-rich protein 2	2	14.90	100.000	100.00	0.0137
MARE1_HUMAN	Microtubule-associated protein RP/EB family member 1	2	7.46	100.000	100.00	0.0186

### TCs *versus* ECs 5th day in cell culture

#### Up-regulated proteins

We identified, by comparison between TCs and ECs, that there are 38 proteins up-regulated in TCs, especially Myosin-14 (18.84-fold), superoxide dismutase (SOD2; 14.59-fold), acid ceramidase (AC; 7.63-fold), envoplakin and epiplakin (∽6-fold each).

#### Down-regulated proteins

In TCs, compared to ECs there are 60 proteins down-regulated, especially cell surface glycoprotein MUC18 (15.54-fold), Ras-interacting protein 1 (13.42-fold), BTB/POZ domain-containing protein (7.26-fold), peptidyl prolyl cis/trans isomerase (6.65-fold) and nestin (5.92-fold) and von Willebrand factor (5.74-fold).

#### Functional analysis of the identified proteins

**Figure 1:**
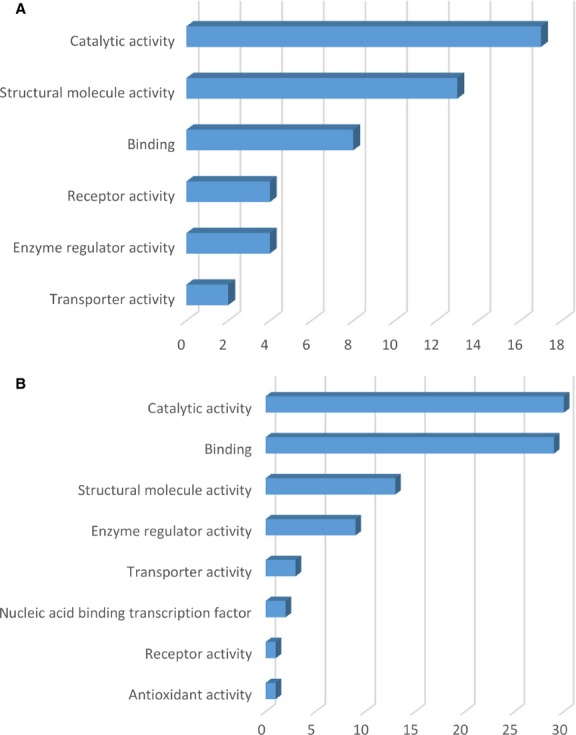
Molecular function classification of proteins found at day 5 in TCs (A) and in ECs (B). Bar graphs based on the PANTHER (Protein ANalysis THrough Evolutionary Relationships) system.

**Figure 2:**
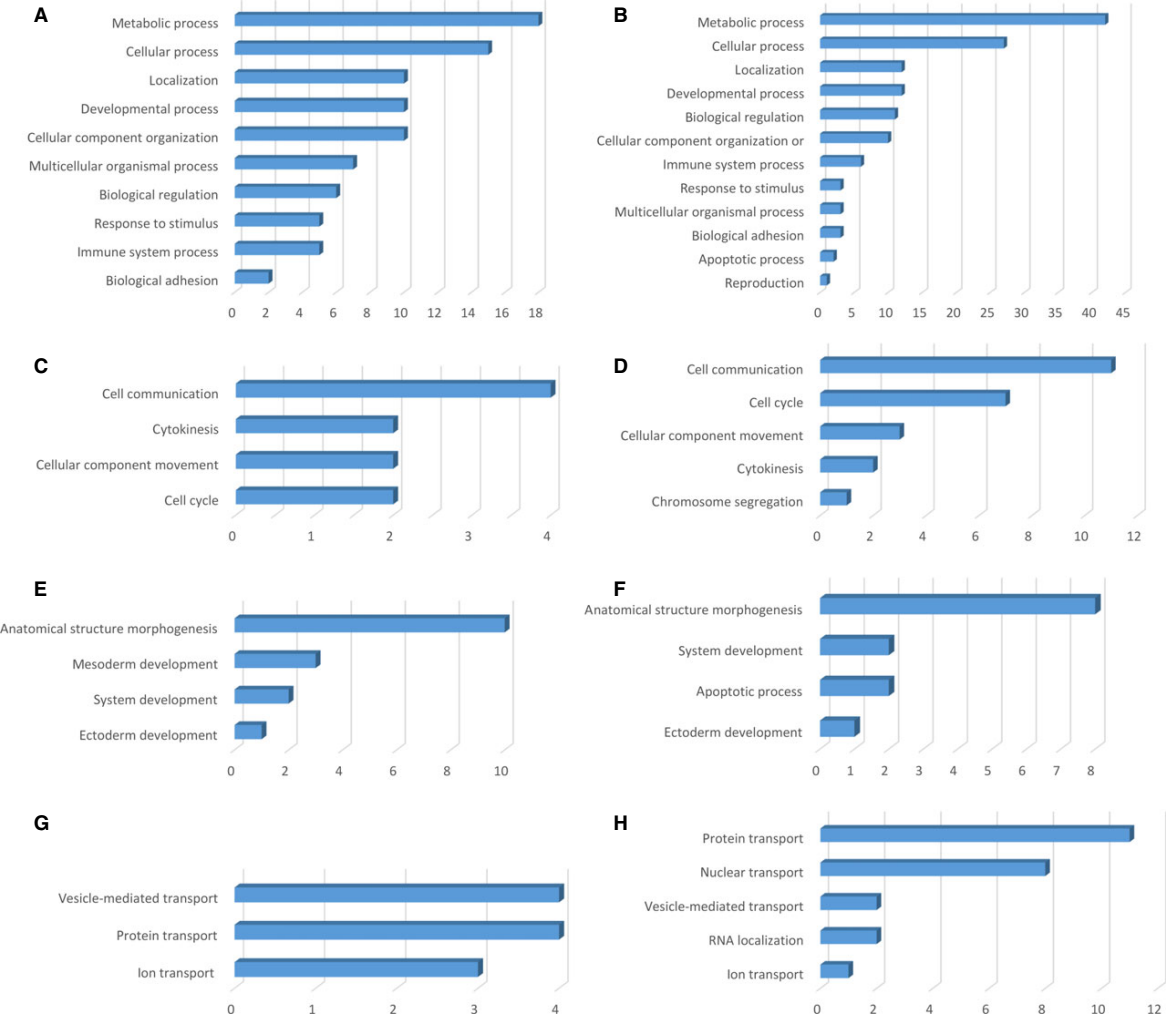
Analysis of differentially expressed proteins at day 5 in TCs *versus* ECs by biological process (A and B), cellular processes (C and D), developmental processes (E and F) and localization (G and H).

**Figure 3:**
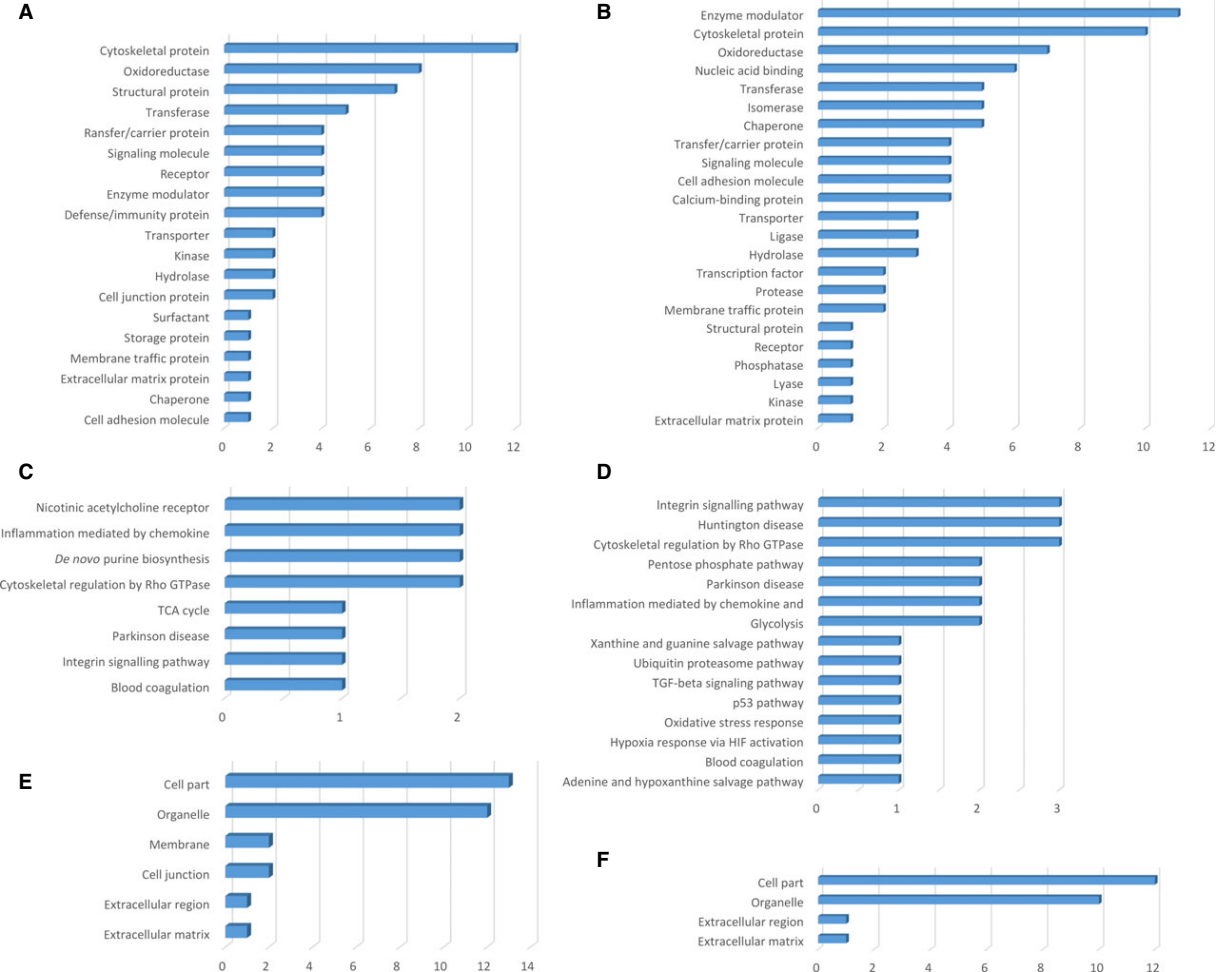
Bar graph representation of the distribution of identified proteins in TCs and ECs (cell culture, 5th day) according to their protein class (A and B), pathways (C and D) and cellular components (E and F) classification.

The protein expression profiles were analysed with the aid of PANTHER Classification System and depicted in Figures1–3 (details are given in Tables S1 and S2). The highly expressed proteins in TCs are involved in important *molecular functions* such as: catalytic activity (17 proteins), structural molecule activity (13 proteins) as seen in Figure1A compared to ECs where significantly more proteins are involved in catalytic activity (30 proteins) and 29 proteins have molecular binding function (Fig.1B). In addition, in ECs, two up-regulated proteins are involved in nucleic acid-binding transcription and one has anti-oxidant activity.

Analysis of PANTHER *biological processes,* in which the 38 up-regulated proteins in TCs are involved, revealed that 18 proteins are responsible for metabolic processes and 15 proteins in *cellular processes* - Figure2A, such as cell communication (4 proteins), cytokinesis (2 proteins), cellular component movement (2 proteins), cell cycle (2 proteins) - Figure2C. Ten up-regulated proteins in TCs are involved in *developmental processes*: anatomical structure morphogenesis (10 proteins), mesoderm development (3 proteins), system development (2 proteins) and ectoderm development (1 protein) - Figure2E. Moreover, 10 proteins in TCs are related to *localization* processes such as vesicle mediated transport (4 proteins), protein transport (4 proteins) and ion transport (3 proteins) - Figure2G.

*Biological processes* of the ECs are related to 42 up-regulated proteins correlated with metabolic processes, 27 to *cellular processes* (Fig.2B) such as cell communication (11 proteins), cell cycle (7 proteins), cellular component movement (3 proteins), cytokinesis (2 proteins), chromosome segregation (1 protein) - Figure2D. There are 12 up-regulated proteins which are part of the *developmental processes* such as anatomical structure morphogenesis (8 proteins), system development (2 proteins), apoptotic processes (2 proteins), ectoderm development (1 protein) - Figure2F. Twelve proteins in ECs participate in *localization* processes *e.g*. protein transport (11 proteins), nuclear transport (8 proteins), vesicle mediated transport (2 proteins), RNA localization (2 proteins) and ion transport (1 protein) - Figure2H.

Interestingly to note, there are 6 up-regulated proteins in TCs compared to ECs involved in biological regulation and 5 related to response to stimulus.

The *protein classes* of the TCs enclose cytoskeletal proteins (12 proteins), oxidoreductase (8 proteins), structural proteins (7 proteins), transferase (5 proteins) *etc*. - Figure3A. The up-regulated TCs proteins belong to the following *pathways*: nicotinic acetylcholine receptor (2 proteins), inflammation mediated by chemokines (2 proteins), *de novo* purine biosynthesis (2 proteins), cytoskeletal regulation by Rho GTPase (2 proteins), TCA cycle (1 protein), Parkinson disease (1 protein), integrin signalling (1 protein) and blood coagulation (1 protein) - Figure3C. In TCs, the up-regulated proteins are related to the following *cellular components*: cell part (13 proteins), organelle (12 proteins), membrane (2 proteins), cell junction (2 proteins), extracellular region (1 protein) and extracellular matrix (1 protein) - Figure3E.

The up-regulated proteins in ECs are attributed to the following *protein classes*: enzyme modulator (11 proteins), cytoskeletal proteins (10 proteins), oxidoreductase (7 proteins), nucleic acid binding (6 proteins), transferase, isomerase and chaperone (5 proteins each), *etc*. - Figure3B. The *pathways* map depicted the ECs proteins are related to: integrin signalling pathway (3 proteins), Huntington disease (3 proteins), cytoskeletal regulation by Rho GTPase (3 proteins), pentose phosphate pathway (2 proteins), Parkinson disease (2 proteins), inflammation mediated by chemokines (2 proteins), glycolysis (2 proteins), *etc*. - Figure3D. The *cellular component* of ECs proteome demonstrated proteins related to: cell part (12 proteins), organelle (10 proteins), extracellular region (1 protein) and extracellular matrix (1 protein) - Figure3F.

**Figure 4:**
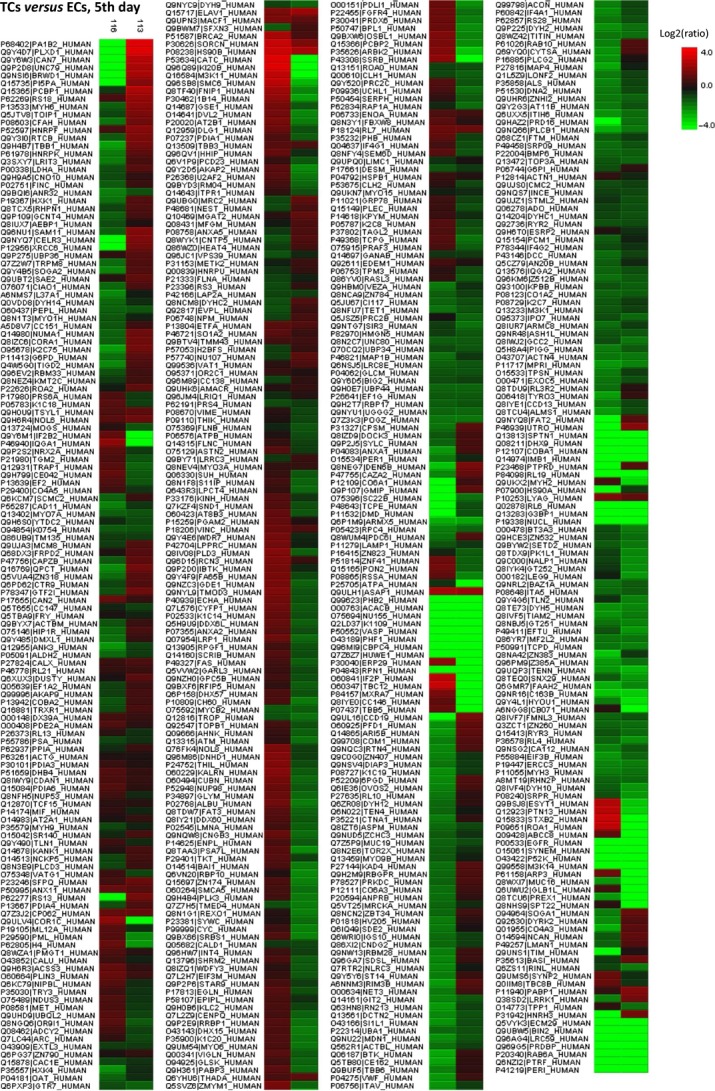
Heat map generated from iTRAQ data by using PEAKS. It shows differential expression results between TCs and ECs (cell culture, 5th day). Experimental samples are clustered on the horizontal axis and protein spots on the vertical axis. Colours correspond to the level of the measurement: red indicates increased and green decreased expression ratio, while black squares indicate no change in protein abundance.

The heat map showing the differentially expressed proteins between TCs and ECs, in cell culture after 5 days, can be observed in Figure4.

### TCs *versus* ECs, 10th day in cell culture

#### Up-regulated proteins

Telocytes as compared to ECs, showed that there are 26 proteins up-regulated in TCs, especially prostacyclin synthase (8.93-fold), epiplakin (4.78-fold) and superoxide dismutase (4.50-fold).

#### Down-regulated proteins

In TCs, compared to ECs there are 56 proteins down-regulated, especially microtubule-associated protein RP/EB family member 1 (100-fold), cysteine-rich protein 2 (100-fold), von Willebrand factor (15.89-fold) and platelet endothelial cell adhesion molecule (13.31-fold) peptidyl prolyl cis/trans isomerase (10.53-fold) and cell surface glycoprotein MUC18 (9.31-fold) - Table2 (For details see Tables S3 and S4).

#### Functional analysis of the identified proteins

**Figure 5:**
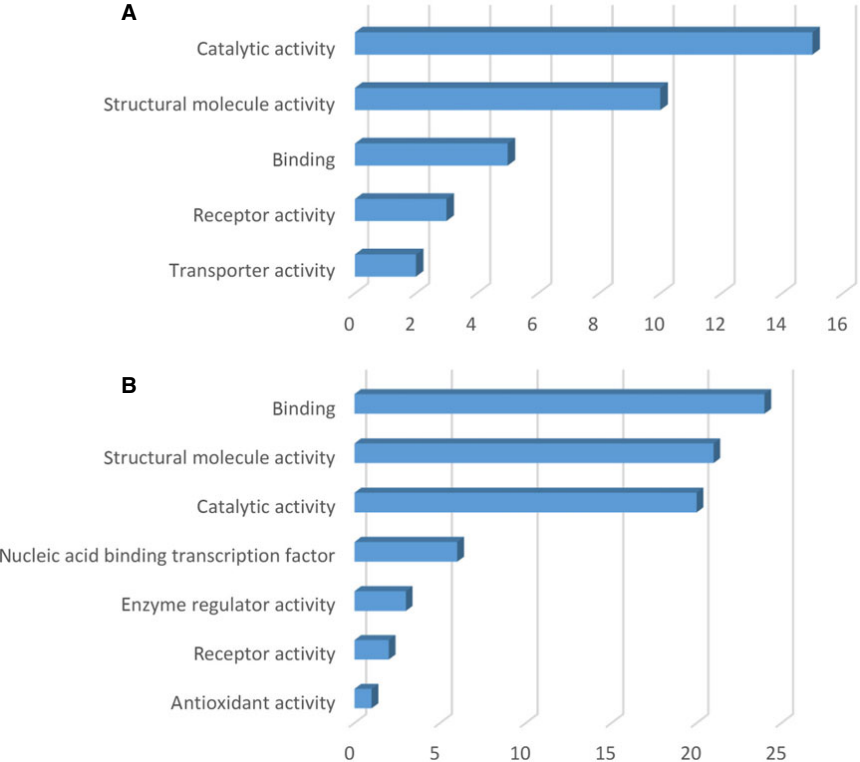
Molecular function classification of proteins found at day 10 in TCs (A) and in ECs (B). Bar graphs based on the PANTHER (Protein ANalysis THrough Evolutionary Relationships) system.

**Figure 6:**
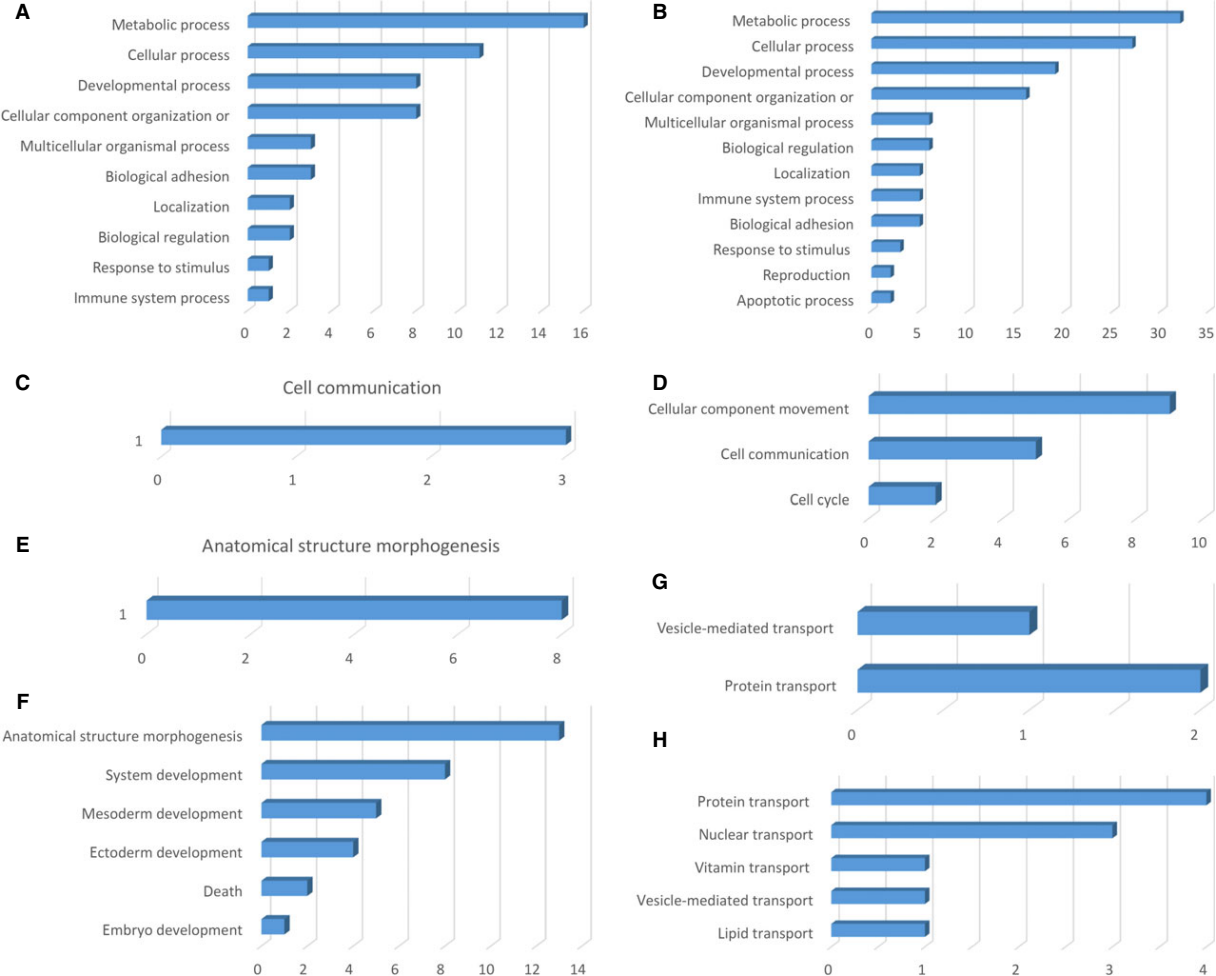
Analysis of differentially expressed proteinsat day 10 in TCs *versus* ECs by biological process (A and B), cellular processes (C and D), developmental processes (E and F) and localization (G and H).

**Figure 7:**
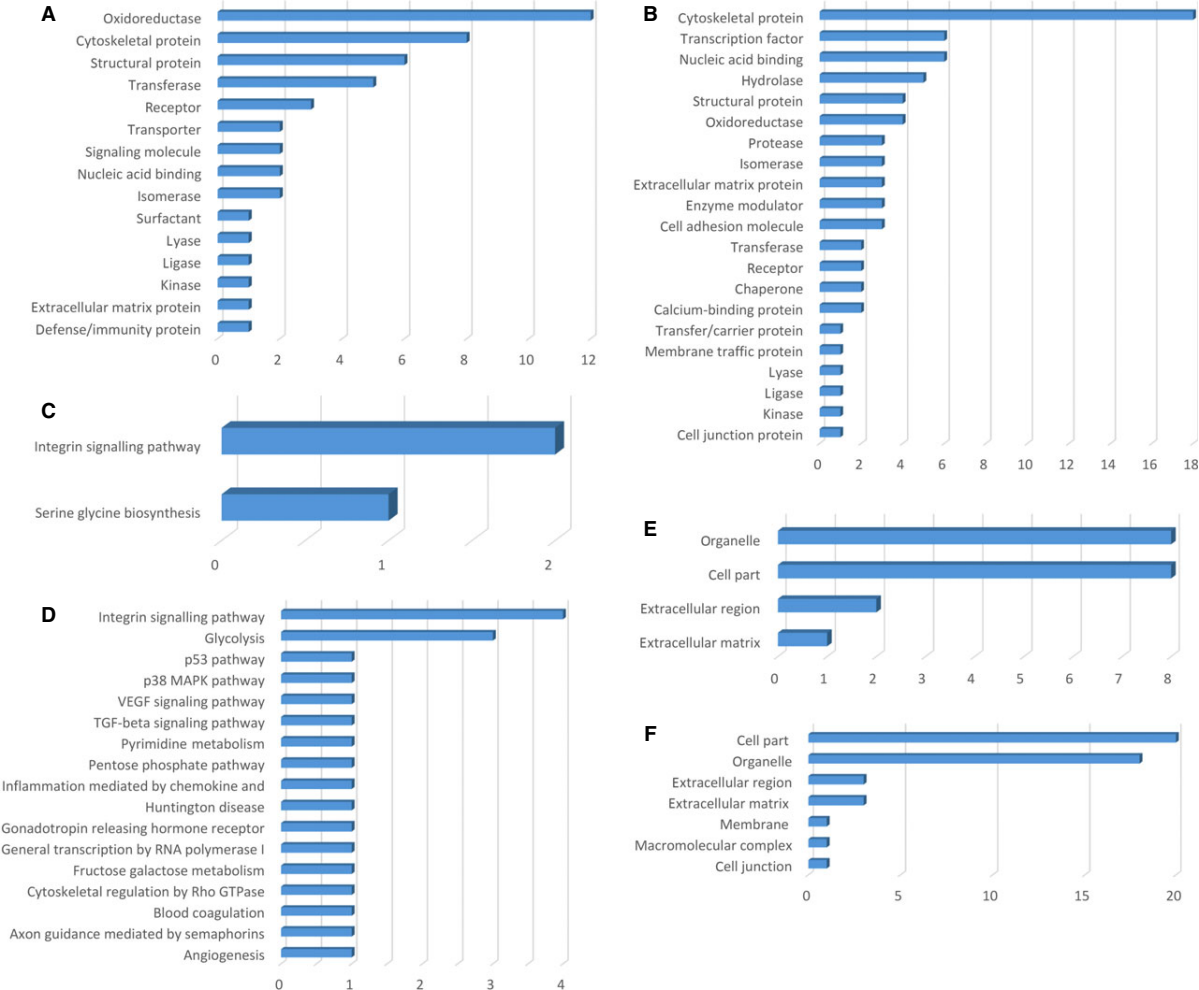
Bar graph representation of the distribution of identified proteins in TCs and ECs (cell culture, 10th day) according to their protein class (A and B), pathways (C and D) and cellular components (E and F) classification.

Figures5–7 show the distributions of differentially proteins in putative functional categories. The highly expressed proteins in TCs are involved in important *molecular functions* such as: catalytic activity (15 proteins), structural molecule activity (10 proteins), binding (5 proteins), receptor activity (3 proteins), transporter activity (2 proteins) as seen in Figure5A compared to ECs where significantly more proteins are involved in binding (24 proteins), structural molecule activity (21 proteins), catalytic activity (20 proteins), nucleic acid-binding transcription (6 proteins), enzyme regulator activity (3 proteins), anti-oxidant activity (1 protein) - Figure5B.

The 26 up-regulated proteins found in TCs were assigned to the following *biological processes* (according to PANTHER): metabolic processes (16 proteins), cellular processes (11 proteins), developmental processes (8 proteins), cellular component organization (8 proteins), *etc*. - (Fig.6A). The only *cellular process* which involve TCs up-regulated proteins is cell communication - Figure6C. Eight up-regulated proteins in TCs are involved in one *developmental process* - anatomical structure morphogenesis - Figure6E. Two proteins in TCs are related to *localization* processes such as vesicle mediated transport (1 protein) and protein transport (2 proteins) - Figure6G.

The 56 proteins found to be up-regulated in the ECs are assigned to the following *biological processes:* metabolic processes (32 proteins), cellular processes (27 proteins), developmental processes (18 proteins) - Figure6B. The *cellular processes* which involve ECs proteins are: cellular component movement (9 proteins), cell communication (5 proteins), cell cycle (2 proteins) - Figure6D. The main developmental processes which involve ECs proteins are: anatomical structure morphogenesis (13 proteins), system development (8 proteins), mesoderm development (5 proteins), ectoderm development (4 proteins), death (2 proteins), embryo development (1 protein) - Figure6F. Five proteins in ECs participate in localization processes *e.g*. protein transport (4 proteins), nuclear transport (3 proteins), vitamin transport (1 protein), vesicle mediated transport (1 protein), lipid transport (1 protein) - Figure6H.

The *protein classes* of the TCs enclose oxidoreductase (12 proteins), cytoskeletal proteins (8 proteins), structural proteins (6 proteins), transferase (5 proteins) *etc*. - Figure7A. The up-regulated TCs proteins belong to the following *pathways*: integrin signalling pathway (2 proteins) serine glycine biosynthesis (1 protein) - Figure7C. In TCs, the up-regulated proteins are related to the following *cellular components*: cell part (8 proteins), organelle (8 proteins), extracellular region (2 proteins) and extracellular matrix (1 protein) - Figure7E.

The up-regulated proteins in ECs are attributed to the following *protein classes*: cytoskeletal proteins (18 proteins), transcription factor (6 proteins), nucleic acid binding (6 proteins), hydrolase (5 proteins), oxidoreductase (4 proteins), structural protein (4 proteins), *etc*. - Figure7B. The *pathways* map depicted the ECs proteins are related to: integrin signalling pathway (4 proteins), glycolysis (2 proteins), *etc*. - Figure7D. The *cellular component* of ECs proteome demonstrated proteins related to: cell part (20 proteins), organelle (18 proteins), extracellular region (3 proteins) and extracellular matrix (3 proteins), membrane (1 protein), macromolecular complex (1 protein), cell junction (1 protein) - Figure7F.

**Figure 8:**
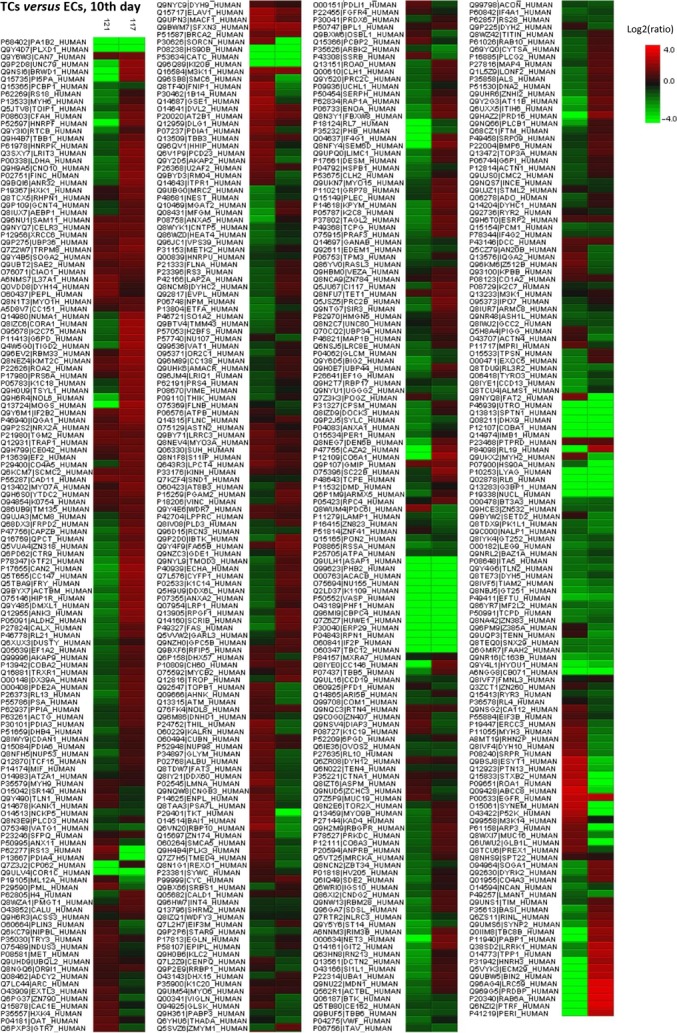
Differentially expressed proteins between TCs and ECs (cell culture, 10th day) were analysed by hierarchical clustering. In the heat map the experimental samples are clustered on the horizontal axis and protein spots on the vertical axis. Red: up-regulation; green: down-regulation; black: no change.

The heat map presenting the differentially expressed proteins between TCs and ECs is showed in Figure8 and demonstrate that the differences between this two cell types are still preserved in cell culture after 10 days.

**Figure 9:**
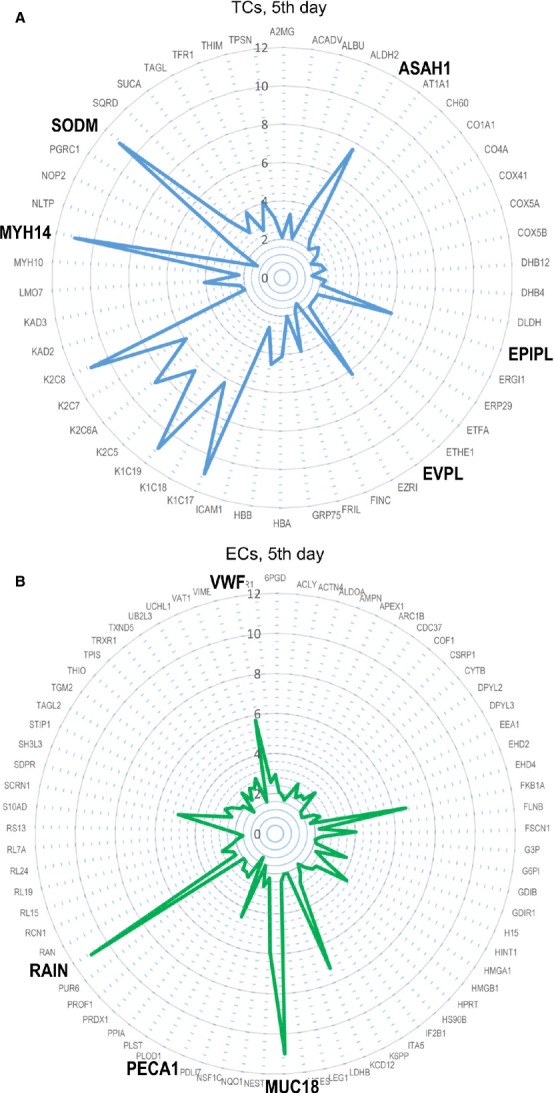
Radar plots of proteomic profile for top proteins of TCs (A) and ECs (B) at 5th day in cell culture.

**Figure 10:**
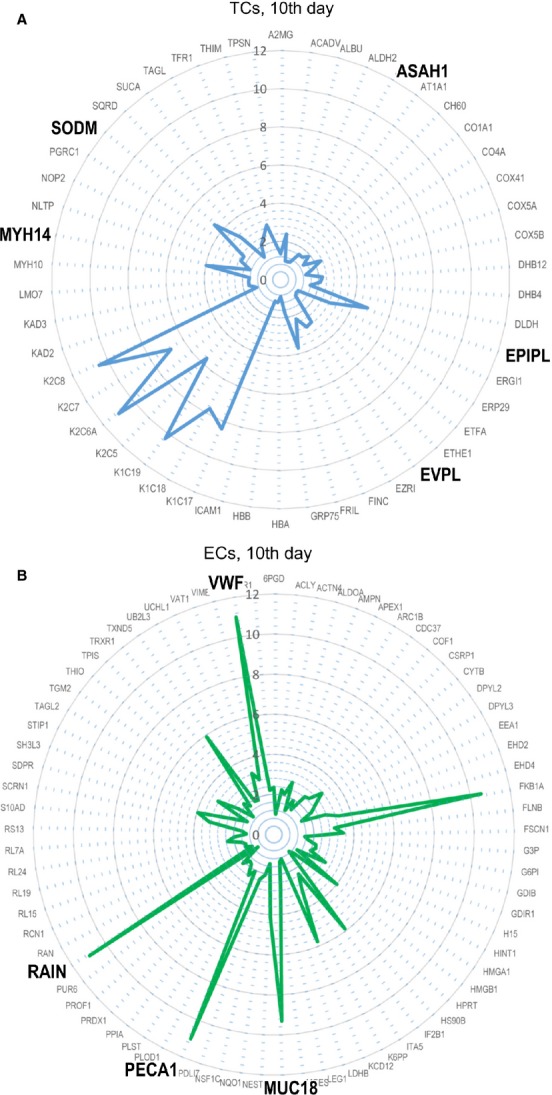
Radar plots of proteomic profile for top proteins of TCs (A) and ECs (B) at 10th day in cell culture.

Figures9 and 10 use radar-chart representation of differentially expressed proteins between TCs and ECs at 5th day and at 10th day in cell cultures. Radar charts were chosen because they allow the visualization of a large numbers of proteins at the same time.

A String Network analysis was also performed to study the relation among differentially expressed proteins. In the global STRING-generated protein-protein network, several complexes and cellular functions formed prominent, tightly connected clusters as assessed by means of molecular complex detection (see Figs S1–S4). Figures1 and 2 quantify protein-interaction properties of the TCs and ECs, respectively where the confidence view is presented and stronger associations are represented by thicker lines. These results indicate that while TCs are involved mainly in oxidation-reduction processes (Fig.1A), ECs are involved (as expected) in haemostasis (Fig.2A). Both cell types release extracellular vesicles (exosomes) [29,49], however their content is different as indicated in Figures1B and 2B.

**Figure 11:**
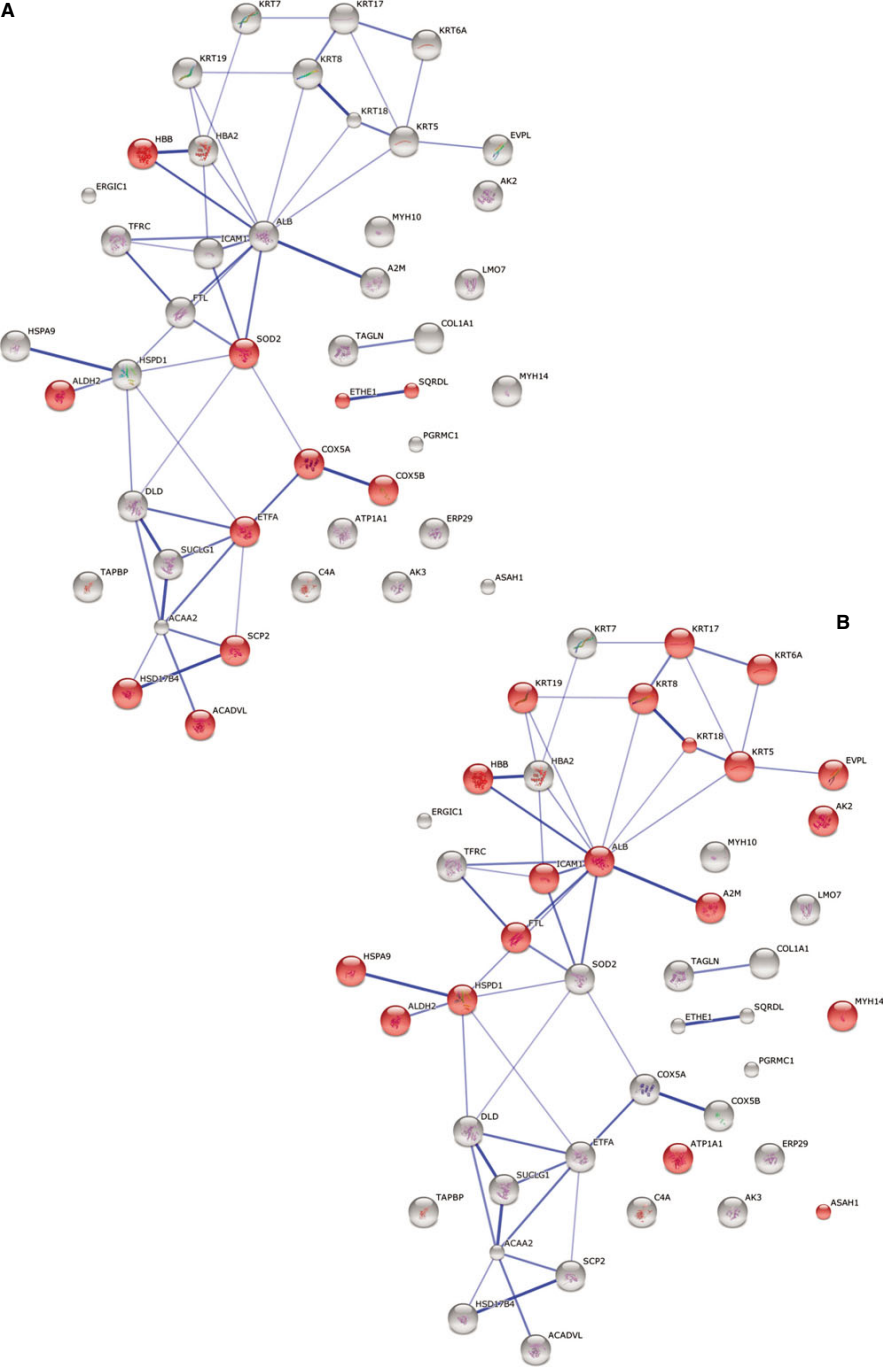
Protein interaction network generated with STRING. Major clusters of interacting proteins include those involved in oxidation-reduction process (A) and extracellular vesicular exosome (B) for TCs at day 5. Red nodes represent up-regulated proteins involved in these processes.

**Figure 12:**
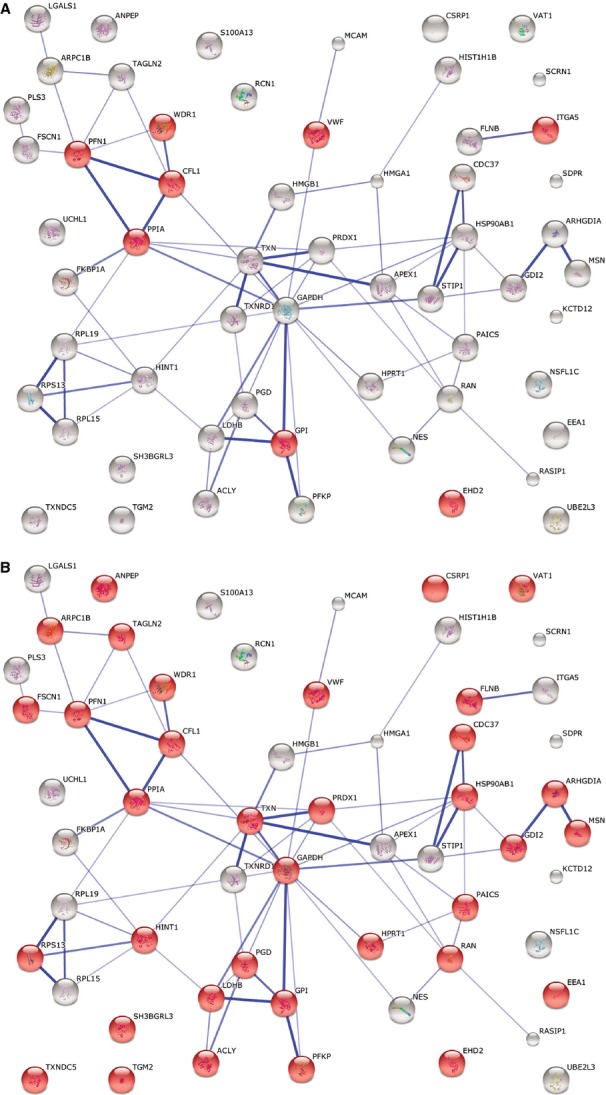
STRING analysis for ECs at day 5 investigating the interactions between up-regulated proteins and depicting ECs involvement in haemostasis (A) and extracellular vesicular exosome (B). Red nodes represent up-regulated proteins involved in these processes.

## Discussion

Previously, we performed a proteomic analysis of human lung TCs compared to fibroblasts, at different time-points (the 5th and 10th day in primary cell culture) and we demonstrated that TCs protein expression profile is different [34]. The results were suggestive for specific roles of TCs in mechanical sensing and mechanic-chemical conversion task, tissue homoeostasis and remodelling/renewal. In addition, the presence of some proteins, specific for extracellular vesicles, emphasize TCs roles in intercellular signalling and stem cell niche modulation [19,34,50].

Beyond scientific interest in general, the comparison of TCs with ECs has a specific purpose. Both TCs and ECs are immunohistochemically CD34 positive, but while ECs are CD31 positive, TCs are CD31 negative. The present proteomic comparison confirms these immunohistochemical differences.

### Putative roles of differentially expressed proteins

We previously showed that **myosin-14** which is the main up-regulated protein in TCs make these cells candidates for a *mechanical sensing and mechanochemical conversion task* [34].

Telocytes proteome revealed the presence of **SOD2 (SODM)**, a tetrameric anti-oxidative enzyme located in the mitochondrial matrix, encoded by genes located on chromosome 6 (6q25.3). The enzyme has manganese in its reactive centre, and catalyse the dismutation of superoxide (O_2_^−^) into oxygen and hydrogen peroxide. SOD2 act as a cytoprotective enzyme proved to be essential for the survival of aerobic organisms [51]. It also serves as *key anti-oxidant* being considered a *tumour suppressor protein via* modulating redox-related transcriptional factors [52].

**Acid ceramidase**, (an enzyme encoded by the ASAH1 gene) which was found to be up-regulated in TCs, is located in lysosomes and active at acidic pH [53]. It was shown to have a noteworthy position in cancer biology: high AC activity leads to an enhanced cell growth, while low AC activity leads to reduced cell growth through an enhanced ceramide response [54]. Also, AC has been shown to play important roles in tumour pathogenesis, and in resistance to therapy having a key role in controlling the ceramide-sphingosine-sphingosine-1-phosphate (S1P) balance that regulates cellular homoeostasis[55]. Therefore, we can hypothesize that TCs might have pro-proliferative effects through the inhibition of apoptosis through the regulation of inter-conversion of ceramide, sphingosine and S1P.

**Envoplakin** is a protein that in humans is encoded by the EVPL gene, and it is a member of a family of large multi-domain molecules [56]. Periplakin (195 kD) and envoplakin (210 kD) are closely related and have various functions to link cytoskeletal elements together and to connect them to junctional complexes. As we previously suggested, the presence of plakins in TCs is related to their homo and heterocellular junctions and it might be related to mechanical sensing and mechanochemical conversion task [34]. Plakins may also have additional roles in signal transduction [56].

Endothelial cells proteomic analysis revealed that proteins like microtubule-associated protein RP/EB family member 1, **MUC18**, **cysteine-rich protein 2**, **von Willebrand factor** (15.89-fold) and **platelet endothelial cell adhesion molecule** were found to be up-regulated at 5th day and also at 10th day in ECs culture. Microtubule-associated protein RP/EB family member 1 is a ubiquitously expressed protein which binds to the plus end of microtubules and regulates the dynamics of the microtubule cytoskeleton, probably playing a role in cell migration [57]. **MUC18 (CD146)** is a glycoprotein detected in endothelial cells as a surface receptor that triggers a transient increase in the intracellular calcium concentration [58]. **Cysteine-rich protein (CRP) 2** is a member of the LIM-only CRP family, also expressed in vascular smooth muscle cells (VSMCs) of blood vessels [59]. Its role is to repress VSMC migration and vascular remodelling, because it was demonstrated that the absence of CRP2 increases neointima formation, correlating with increased VSMC migration [60]. **von Willebrand factor** is a haemostatic protein stored in Weibel Palade bodies (considered as a hallmark of endothelial cells) until release [61]. In addition, we identified Ras-interacting protein 1 (RAIN) as being overexpressed in ECs. We can consider RAIN - known to be essential for endothelial cell morphogenesis and blood vessel tubulogenes - as being a part of the specific signature for ECs, in consistency with other recent proteomic study [62].

We found no significant differences between protein expression, in ECs, at 5 days and at 10 days.

Our present results suggest that TCs are cells relatively rich in mitochondria, which correlates with previous findings [34]. The primary functions of mitochondria include: generating energy by oxidative phosphorylation, creating reactive oxygen species (ROS) and regulating apoptosis. It is also known that cellular ageing is influenced by oxidative phosphorylation, ROS and telomeres. Therefore, this study enabled us to suggest TCs involvement in the modulation of oxidative stress levels which might lead to a rigorous control in apoptosis activation. This finding is also in agreement with the fact that TCs are decreasing during ageing of myocardium (work in progress).

This study provides a comprehensive approach to analyse the comparative proteome between TCs and ECs and we can conclude that the significant discriminative power of each of the proteins mentioned above supports the case for TCs as distinctive cells, while ECs are characterized by the already known marker molecules such as MUC18 and von Willebrand factor. Also, it supports once more the idea of TCs involvement in tissue homoeostasis and in stem cell activity, as previously suggested by our group.

Moreover, it stands for a recent perspective suggested by Smithies and Edelstein considering TCs as a very primitive nervous system at the cellular level which can play a major role in morphogenetic bioelectrical signalling [63,64].

In conclusion, the current proteomic analysis presented here, clearly depicts that TCs are completely different from ECs. Protein expression profile demonstrates that TCs might play specific roles in intercellular signaling and also as physical and/or chemical sensors. Their close relationships with stem cells should not be overlooked.

## Supporting Information

**Figure S1** STRING analysis of up-regulated proteins in TCs at day 5 representing the confidence view where stronger associations are represented by thicker lines.

**Figure S2** STRING analysis of up-regulated proteins in ECs at day 5 representing the confidence view where stronger associations are represented by thicker lines.

**Figure S3** STRING analysis of up-regulated proteins in TCs at day 10 representing the confidence view where stronger associations are represented by thicker lines.

**Figure S4** STRING analysis of up-regulated proteins in ECs at day 10 representing the confidence view where stronger associations are represented by thicker lines.

**Table S1** Summary of up-regulated TCs proteins (more than twofold) versus ECs 5th day. **Table S2** Summary of up-regulated ECs proteins (more than twofold) versus TCs 5th day. **Table S3** Summary of up-regulated TCs proteins (more than twofold) versus ECs 10th day. **Table S4** Summary of up-regulated ECs proteins (more than twofold) versus TCs 10th day.
