# Supplementary figures and images for: Protein profiling of human lung telocytes and microvascular endothelial cells using iTRAQ quantitative proteomics
Source: J Cell Mol Med. 2014 Jul 24;18(6):1035–59. doi: 10.1111/jcmm.12350 (PMC4508144; doi:10.1111/jcmm.12350)

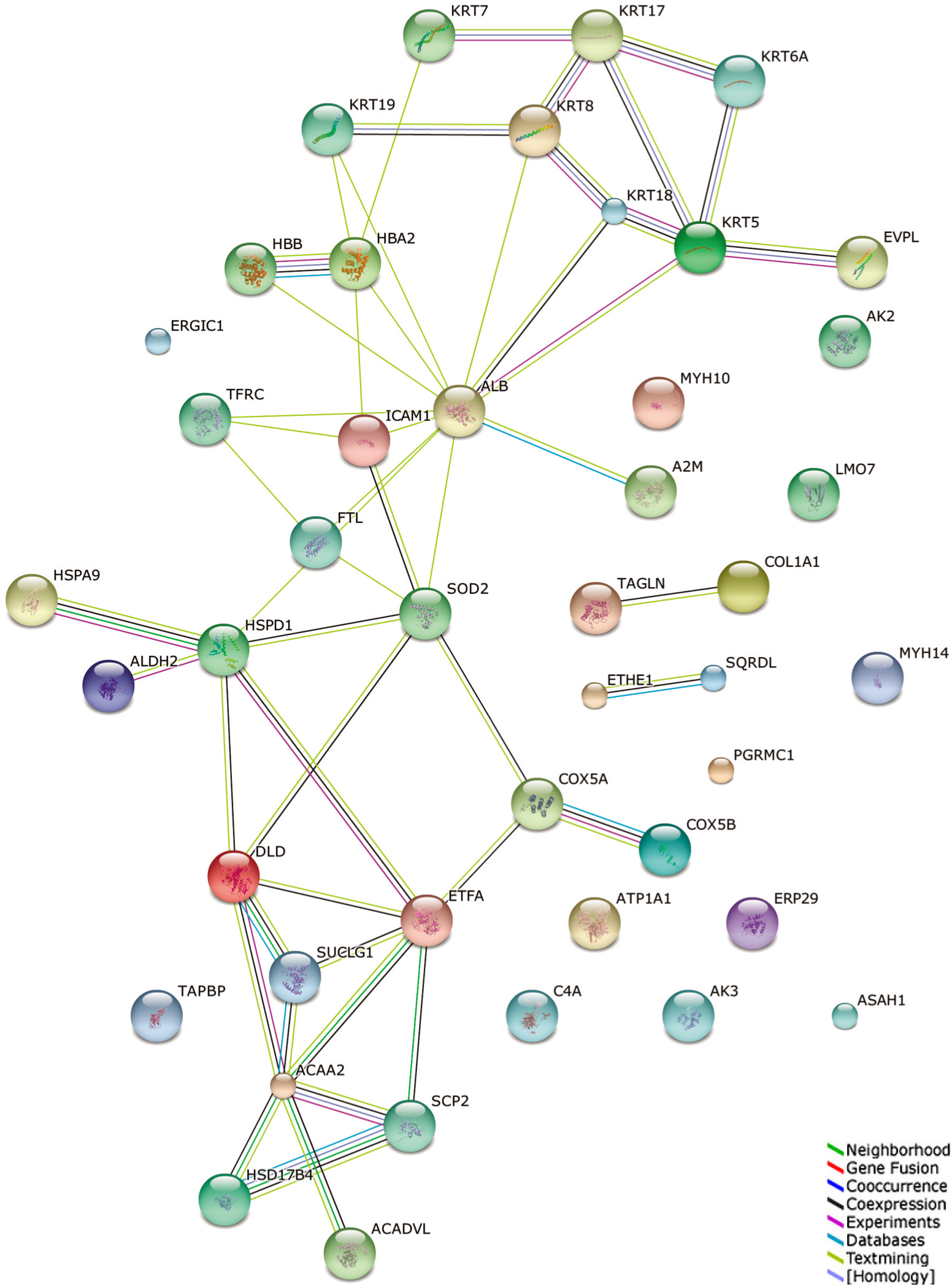

Supplement: Supplementary file 1 [file jcmm0018-1035-sd1.tif]

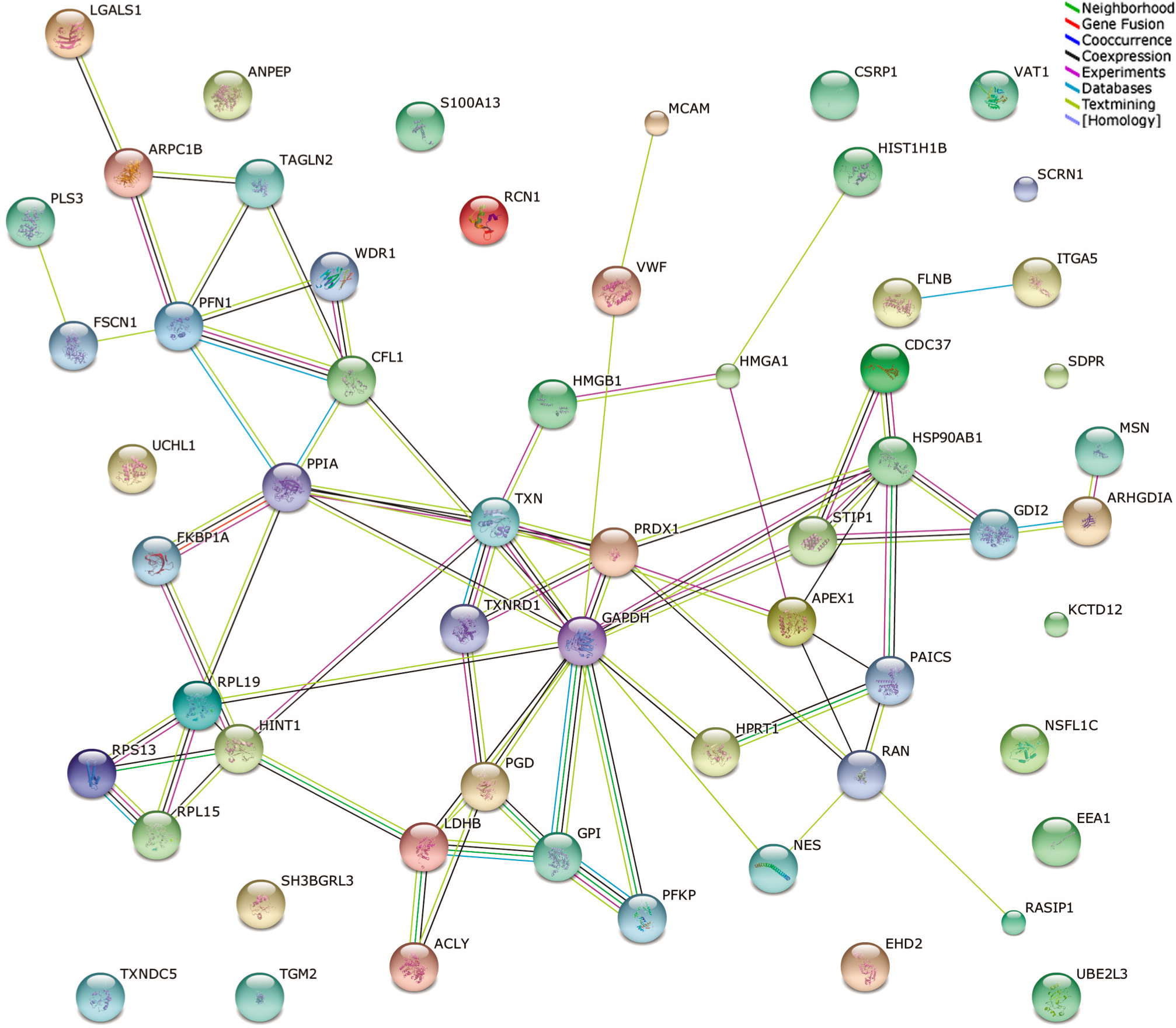

Supplement: Supplementary file 3 [file jcmm0018-1035-sd3.tif]

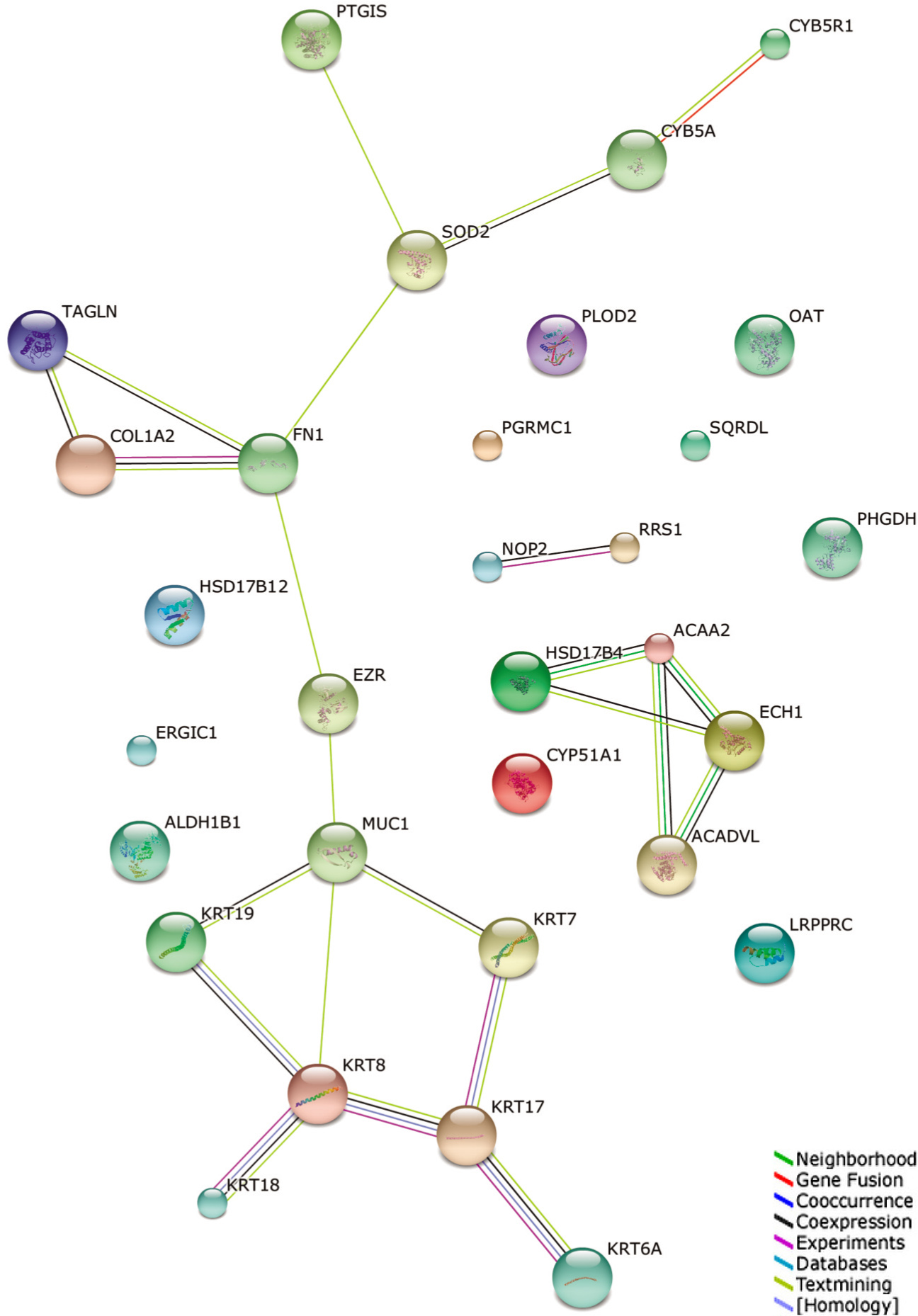

Supplement: Supplementary file 4 [file jcmm0018-1035-sd4.tif]

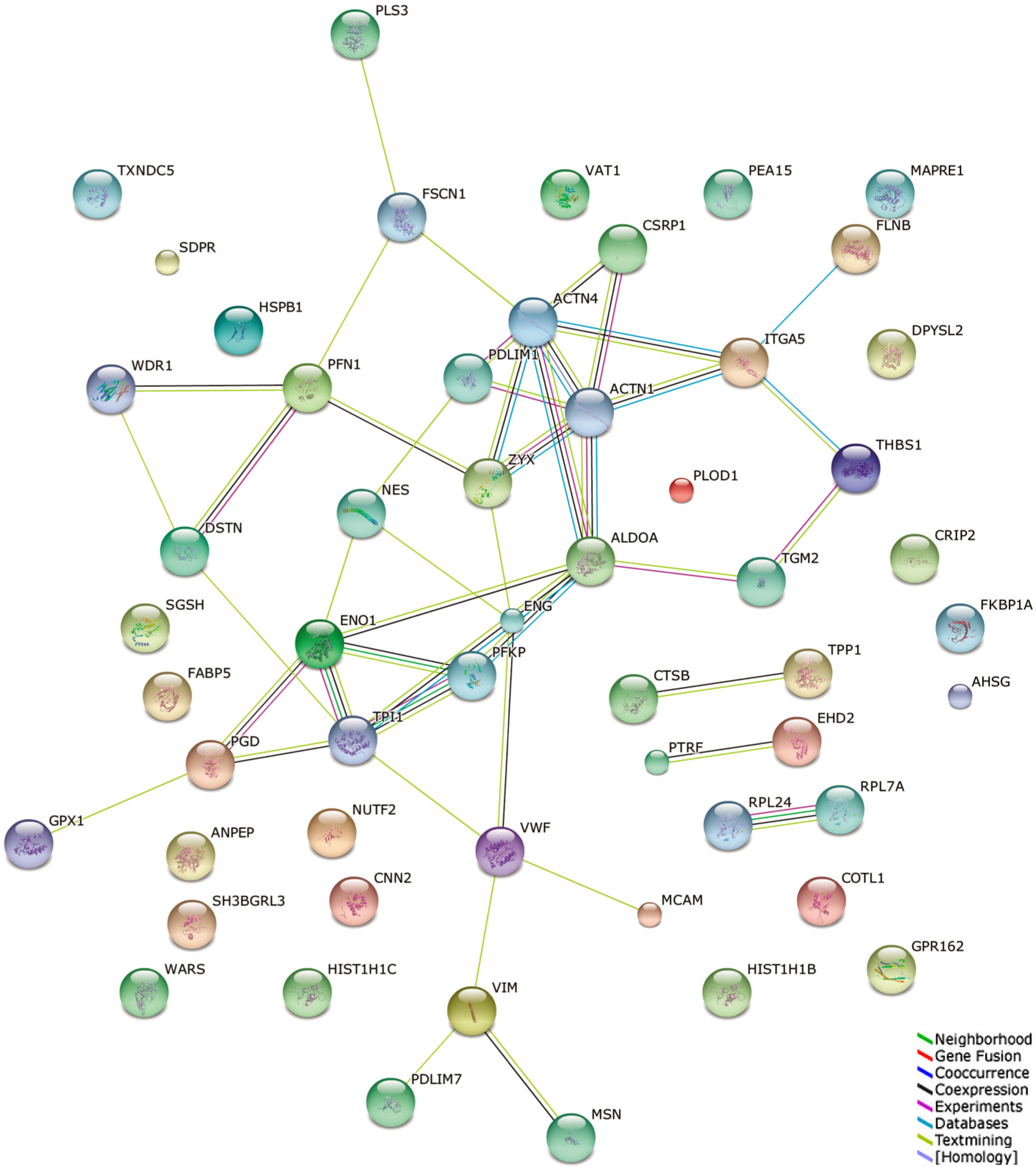

Supplement: Supplementary file 5 [file jcmm0018-1035-sd5.tif]
