# Supplementary material for: Protein profiling of human lung telocytes and microvascular endothelial cells using iTRAQ quantitative proteomics
Source: J Cell Mol Med. 2014 Jul 24;18(6):1035–59. doi: 10.1111/jcmm.12350 (PMC4508144; doi:10.1111/jcmm.12350)
Supplement: Supplementary file 2 [file jcmm0018-1035-sd2.docx]

Supplementary table 1. TC5

| Nr.crt. | Gene Symbol | Gene Name | Molecular Function | Biological Process | Cellular Component | Protein Class | Pathway |
| --- | --- | --- | --- | --- | --- | --- | --- |
| 1. | A2MG | Alpha-2-macroglobulin | peptidase activity; cytokine activity; serine-type endopeptidase inhibitor activity | complement activation; proteolysis; cellular process; response to stimulus; regulation of catalytic activity | - | cytokine; serine protease inhibitor; complement component | Blood coagulation->alpha-2-macroglobulin |
| 2. | ACADV | Very long-chain specific acyl-CoA dehydrogenase, mitochondrial | oxidoreductase activity; transferase activity | respiratory electron transport chain; acyl-CoA metabolic process; nitrogen compound metabolic process; fatty acid beta-oxidation; acyl-CoA metabolic process | - | transferase; dehydrogenase; oxidase | - |
| 3. | ALBU | Serum albumin | - | transport | - | transfer/carrier protein | - |
| 4. | ALDH2 | Aldehyde dehydrogenase, mitochondrial | oxidoreductase activity | nucleobase-containing compound metabolic process; cellular amino acid metabolic process | - | dehydrogenase | - |
| 5. | ASAH1 | Acid ceramidase | - | - | - | - | - |
| 6. | AT1A1 | Sodium/potassium-transporting ATPase subunit alpha-1 | hydrolase activity; ion channel activity; cation transmembrane transporter activity | metabolic process; cation transport; cellular calcium ion homeostasis | - | cation transporter; ion channel; hydrolase | - |
| 7. | CH60 | 60 kDa heat shock protein, mitochondrial | - | protein folding | - | chaperonin | - |
| 8. | CO1A1 | Collagen alpha-1(I) chain | receptor activity; extracellular matrix structural constituent; transmembrane transporter activity | macrophage activation; cell communication; cell-cell adhesion; blood circulation; ectoderm development; mesoderm development; cellular component morphogenesis; response to stimulus; intracellular protein transport; receptor-mediated endocytosis; regulation of liquid surface tension; cellular component organization | extracellular region; extracellular matrix | transporter; surfactant; receptor; extracellular matrix structural protein; antibacterial response protein | Integrin signalling pathway->Collagen |
| 9. | CO4A | Complement C4-A | - | - | - | - | - |
| 10. | CO4A | Complement C4-A | - | - | - | - | - |
| 11. | CO4A | Complement C4-A | peptidase activity; cytokine activity; serine-type endopeptidase inhibitor activity | complement activation; proteolysis; cellular process; response to stimulus; regulation of catalytic activity | - | cytokine; serine protease inhibitor; complement component | - |
| 12. | COX5A | Cytochrome c oxidase subunit 5A, mitochondrial | oxidoreductase activity | oxidative phosphorylation; respiratory electron transport chain | - | oxidase | - |
| 13. | COX5B | Cytochrome c oxidase subunit 5B, mitochondrial | oxidoreductase activity | oxidative phosphorylation; respiratory electron transport chain | mitochondrion; cytoplasm | oxidase | - |
| 14. | DHB4 | Peroxisomal multifunctional enzyme type 2 | oxidoreductase activity | cellular amino acid biosynthetic process; steroid metabolic process | - | dehydrogenase; reductase | - |
| 15. | DLDH | Dihydrolipoyl dehydrogenase, mitochondrial | oxidoreductase activity | respiratory electron transport chain; nitrogen compound metabolic process; ferredoxin metabolic process | - | dehydrogenase; oxidase; reductase | - |
| 16. | ERGI1 | Endoplasmic reticulum-Golgi intermediate compartment protein 1 | - | - | - | - | - |
| 17. | ERP29 | Endoplasmic reticulum resident protein 29 | - | intracellular protein transport; exocytosis | - | membrane traffic protein | - |
| 18. | ETFA | Electron transfer flavoprotein subunit alpha, mitochondrial | oxidoreductase activity; transferase activity | respiratory electron transport chain; acyl-CoA metabolic process; nitrogen compound metabolic process; fatty acid beta-oxidation; acyl-CoA metabolic process | - | transferase; dehydrogenase; oxidase | - |
| 19. | ETHE1 | Protein ETHE1, mitochondrial | hydrolase activity | metabolic process; response to toxic substance | - | hydrolase | - |
| 20. | EVPL | Envoplakin | structural constituent of cytoskeleton; intermediate filament binding | cellular process | intracellular | intermediate filament binding protein | - |
| 21. | FRIL | Ferritin light chain | - | cation transport | - | storage protein | - |
| 22. | GRP75 | Stress-70 protein, mitochondrial | - | - | - | - | Parkinson disease->Heat shock protein 70 |
| 23. | HBA | Hemoglobin subunit alpha | - | blood circulation; transport | - | transfer/carrier protein | - |
| 24. | HBA | Hemoglobin subunit alpha | - | - | - | - | - |
| 25. | HBB | Hemoglobin subunit beta | - | blood circulation; transport | - | transfer/carrier protein | - |
| 26. | ICAM1 | Intercellular adhesion molecule 1 | receptor binding | immune system process; cellular process; cell adhesion | - | signaling molecule; immunoglobulin superfamily cell adhesion molecule | - |
| 27. | K1C17 | Keratin, type I cytoskeletal 17 | structural constituent of cytoskeleton | cellular process; cellular component morphogenesis; cellular component organization | intermediate filament cytoskeleton; intracellular | structural protein; intermediate filament | - |
| 28. | K1C18 | Keratin, type I cytoskeletal 18 | structural constituent of cytoskeleton | cellular process; cellular component morphogenesis; cellular component organization | intermediate filament cytoskeleton; intracellular | structural protein; intermediate filament | - |
| 29. | K1C19 | Keratin, type I cytoskeletal 19 | structural constituent of cytoskeleton | cellular process; cellular component morphogenesis; cellular component organization | intermediate filament cytoskeleton; intracellular | structural protein; intermediate filament | - |
| 30. | K2C5 | Keratin, type II cytoskeletal 5 | structural constituent of cytoskeleton | cellular process; cellular component morphogenesis; cellular component organization | intermediate filament cytoskeleton; intracellular | structural protein; intermediate filament | - |
| 31. | K2C6A | Keratin, type II cytoskeletal 6A | structural constituent of cytoskeleton | cellular process; cellular component morphogenesis; cellular component organization | intermediate filament cytoskeleton; intracellular | structural protein; intermediate filament | - |
| 32. | K2C7 | Keratin, type II cytoskeletal 7 | structural constituent of cytoskeleton | cellular process; cellular component morphogenesis; cellular component organization | intermediate filament cytoskeleton; intracellular | structural protein; intermediate filament | - |
| 33. | K2C8 | Keratin, type II cytoskeletal 8 | structural constituent of cytoskeleton | cellular process; cellular component morphogenesis; cellular component organization | intermediate filament cytoskeleton; intracellular | structural protein; intermediate filament | - |
| 34. | KAD2 | Adenylate kinase 2, mitochondrial | nucleotide kinase activity | purine nucleobase metabolic process; pyrimidine nucleobase metabolic process | - | nucleotide kinase; nucleotide kinase | De novo purine biosynthesis->Adenylate kinase |
| 35. | KAD3 | GTP:AMP phosphotransferase, mitochondrial | nucleotide kinase activity | purine nucleobase metabolic process; pyrimidine nucleobase metabolic process | - | nucleotide kinase; nucleotide kinase | De novo purine biosynthesis->Adenylate kinase |
| 36. | LMO7 | LIM domain only protein 7 | structural constituent of cytoskeleton | muscle contraction | actin cytoskeleton; intracellular | actin family cytoskeletal protein | - |
| 37. | MYH10 | Myosin-10 | motor activity; structural constituent of cytoskeleton; protein binding; enzyme regulator activity | metabolic process; cytokinesis; cellular component movement; mitosis; cell communication; muscle contraction; sensory perception of sound; sensory perception; mesoderm development; cellular component morphogenesis; muscle organ development; intracellular protein transport; vesicle-mediated transport; regulation of catalytic activity; cellular component organization | plasma membrane; cell junction; actin cytoskeleton; intracellular | G-protein modulator; actin binding motor protein; cell junction protein | Inflammation mediated by chemokine and cytokine signaling pathway->Myosin; Nicotinic acetylcholine receptor signaling pathway->Myosin; Cytoskeletal regulation by Rho GTPase->Myosin light chain |
| 38. | MYH14 | Myosin-14 | motor activity; structural constituent of cytoskeleton; protein binding; enzyme regulator activity | metabolic process; cytokinesis; cellular component movement; mitosis; cell communication; muscle contraction; sensory perception of sound; sensory perception; mesoderm development; cellular component morphogenesis; muscle organ development; intracellular protein transport; vesicle-mediated transport; regulation of catalytic activity; cellular component organization | plasma membrane; cell junction; actin cytoskeleton; intracellular | G-protein modulator; actin binding motor protein; cell junction protein | Inflammation mediated by chemokine and cytokine signaling pathway->Myosin; Nicotinic acetylcholine receptor signaling pathway->Myosin; Cytoskeletal regulation by Rho GTPase->Myosin light chain |
| 39. | NLTP | Non-specific lipid-transfer protein | - | - | - | transfer/carrier protein | - |
| 40. | PGRC1 | Membrane-associated progesterone receptor component 1 | receptor activity; receptor binding | cell communication | - | signaling molecule; receptor | - |
| 41. | SODM | Superoxide dismutase [Mn], mitochondrial | oxidoreductase activity | metabolic process | - | oxidoreductase | - |
| 42. | SQRD | Sulfide:quinone oxidoreductase, mitochondrial | - | - | - | - | - |
| 43. | SUCA | Succinyl-CoA ligase [ADP/GDP-forming] subunit alpha, mitochondrial | - | - | - | - | TCA cycle->Succinyl CoA Synthetase |
| 44. | TAGL | Transgelin | structural constituent of cytoskeleton; actin binding | muscle contraction | actin cytoskeleton; intracellular | non-motor actin binding protein | - |
| 45. | TFR1 | Transferrin receptor protein 1 | receptor activity | ion transport | - | receptor | - |
| 46. | THIM | 3-ketoacyl-CoA thiolase, mitochondrial | acetyltransferase activity | protein acetylation | - | acetyltransferase | - |
| 47. | TPSN | Tapasin | receptor activity | antigen processing and presentation; cellular defense response | - | immunoglobulin receptor superfamily; immunoglobulin receptor superfamily | - |
| 48. | TPSN | Tapasin | - | - | - | - | - |

Supplementary table 2. EC5

| Nr.crt. | Gene Symbol | Gene Name | Molecular Function | Biological Process | Cellular Component | Protein Class | Pathway |
| --- | --- | --- | --- | --- | --- | --- | --- |
| 1. | 6PGD | 6-phosphogluconate dehydrogenase, decarboxylating; PGD | oxidoreductase activity | pentose-phosphate shunt | - | dehydrogenase | Pentose phosphate pathway->Gluconate Dehydrogenase |
| 2. | ACLY | ATP-citrate synthase; ACLY | transferase activity; lyase activity; ligase activity | generation of precursor metabolites and energy; coenzyme metabolic process; carbohydrate metabolic process; tricarboxylic acid cycle; lipid metabolic process | - | transferase; lyase; ligase | - |
| 3. | ACLY | ATP-citrate synthase; ACLY | - | - | - | - | - |
| 4. | AMPN | Aminopeptidase N; ANPEP | metallopeptidase activity | proteolysis | - | metalloprotease; metalloprotease | - |
| 5. | APEX1 | DNA-(apurinic or apyrimidinic site) lyase; APEX1 | - | - | - | - | - |
| 6. | ARC1B | Actin-related protein 2/3 complex subunit 1B; ARPC1B | structural constituent of cytoskeleton | cellular process; cellular component morphogenesis; cellular component organization | actin cytoskeleton; intracellular | actin family cytoskeletal protein | Inflammation mediated by chemokine and cytokine signaling pathway->Actin related protein 2/3 complex; Integrin signalling pathway->Actin related protein 2/3 complex; Huntington disease->Actin related protein 2/3 complex; Cytoskeletal regulation by Rho GTPase->Actin related protein 2/3 complex |
| 7. | CDC37 | Hsp90 co-chaperone Cdc37; CDC37 | kinase activity; protein binding; kinase activator activity; kinase regulator activity | protein folding; cytokinesis; mitosis; cell communication; cellular component morphogenesis; regulation of catalytic activity; cellular component organization | - | kinase activator; chaperone | - |
| 8. | CDC37 | Hsp90 co-chaperone Cdc37-like 1; CDC37L1 | kinase activity; protein binding; kinase activator activity; kinase regulator activity | protein folding; cytokinesis; mitosis; cell communication; cellular component morphogenesis; regulation of catalytic activity; cellular component organization | - | kinase activator; chaperone | - |
| 9. | COF1 | Cofilin-1; CFL1 | structural constituent of cytoskeleton; actin binding | protein complex assembly; cellular process; cellular component morphogenesis; cellular component organization; protein complex biogenesis | actin cytoskeleton; intracellular | non-motor actin binding protein | Cytoskeletal regulation by Rho GTPase->Cofilin |
| 10. | CSRP1 | Cysteine and glycine-rich protein 1; CSRP1 | structural constituent of cytoskeleton | immune system process; muscle organ development | actin cytoskeleton; intracellular | actin family cytoskeletal protein | - |
| 11. | EEA1 | Early endosome antigen 1; EEA1 | - | - | - | - | - |
| 12. | EHD2 | EH domain-containing protein 2; EHD2 | catalytic activity; calcium ion binding; protein binding; small GTPase regulator activity | metabolic process; synaptic transmission; neurotransmitter secretion; intracellular protein transport; endocytosis; regulation of catalytic activity | - | membrane traffic protein; G-protein modulator; calcium-binding protein | - |
| 13. | FKB1A | Peptidyl-prolyl cis-trans isomerase FKBP1A; FKBP1A | isomerase activity; binding | cellular protein modification process; cellular process | cytoplasm; neuron projection | isomerase; chaperone; calcium-binding protein | TGF-beta signaling pathway->FK506 binding protein 12KDa |
| 14. | FLNB | Filamin-B; FLNB | structural constituent of cytoskeleton; actin binding | cellular component movement; cellular component morphogenesis; cellular component organization | actin cytoskeleton; intracellular | non-motor actin binding protein | Integrin signalling pathway->Filamin |
| 15. | FSCN1 | Fascin; FSCN1 | structural constituent of cytoskeleton; actin binding | cellular component movement | actin cytoskeleton; intracellular | non-motor actin binding protein | - |
| 16. | G3P | Glyceraldehyde-3-phosphate dehydrogenase; GAPDH | oxidoreductase activity | glycolysis; glycolysis | - | dehydrogenase | Glycolysis->Glyceraldehyde 3-phosphate dehydrogenase; Huntington disease->Glyceraldehyde-3-phosphate dehydrogenase |
| 17. | G6PI | Glucose-6-phosphate isomerase; GPI | isomerase activity | glycolysis; gluconeogenesis; glycolysis | - | isomerase | Pentose phosphate pathway->Glucose-P-Isomerase; Glycolysis->Phosphoglucose isomerase |
| 18. | GDIB | Rab GDP dissociation inhibitor beta; GDI2 | transferase activity, transferring acyl groups; protein binding; small GTPase regulator activity | metabolic process; synaptic transmission; neurotransmitter secretion; intracellular protein transport; vesicle-mediated transport; regulation of catalytic activity | - | acyltransferase; G-protein modulator | - |
| 19. | GDIR1 | Rho GDP-dissociation inhibitor 1; ARHGDIA | catalytic activity; receptor binding; small GTPase regulator activity | metabolic process; cell communication; regulation of catalytic activity | - | signaling molecule; G-protein modulator | - |
| 20. | H15 | Histone H1.5; HIST1H1B | DNA binding | nucleobase-containing compound metabolic process; cellular process; chromatin organization | - | histone | - |
| 21. | HINT1 | Histidine triad nucleotide-binding protein 1; HINT1 | nucleotide phosphatase activity; nucleotide phosphatase activity | metabolic process | - | nucleotide phosphatase; nucleotide phosphatase | - |
| 22. | HMGA1 | High mobility group protein HMG-I/HMG-Y; HMGA1 | double-stranded DNA binding | transcription from RNA polymerase II promoter; mitosis; chromosome segregation; regulation of transcription from RNA polymerase II promoter | - | DNA binding protein | - |
| 23. | HMGB1 | High mobility group protein B1; HMGB1 | sequence-specific DNA binding transcription factor activity; sequence-specific DNA binding transcription factor activity; chromatin binding; receptor binding | transcription from RNA polymerase II promoter; cell communication; regulation of transcription from RNA polymerase II promoter; chromatin organization | - | HMG box transcription factor; signaling molecule; chromatin/chromatin-binding protein | p53 pathway->High mobility group protein 1 |
| 24. | HPRT | Hypoxanthine-guanine phosphoribosyltransferase; HPRT1 | transferase activity, transferring glycosyl groups; binding | phosphate-containing compound metabolic process; nitrogen compound metabolic process; biosynthetic process; purine nucleobase metabolic process; cellular process | cytoplasm | glycosyltransferase; mutase | Xanthine and guanine salvage pathway->Guanine phosphoribosyltransferase; Adenine and hypoxanthine salvage pathway->Hypoxanthine phosphoribosyl transferase |
| 25. | HS90B | Heat shock protein HSP 90-beta; HSP90AB1 | - | immune system process; protein folding; response to stress | - | Hsp90 family chaperone | - |
| 26. | ITA5 | Integrin alpha-5; ITGA5 | - | cellular process; cell adhesion | - | cell adhesion molecule | Integrin signalling pathway->Integrin alpha |
| 27. | K6PP | 6-phosphofructokinase type C; PFKP | carbohydrate kinase activity | glycolysis; glycolysis | - | carbohydrate kinase; carbohydrate kinase | - |
| 28. | KCD12 | BTB/POZ domain-containing protein KCTD12; KCTD12 | protein binding | cation transport | - | enzyme modulator | - |
| 29. | LDHB | L-lactate dehydrogenase B chain; LDHB | oxidoreductase activity | glycolysis; glycolysis; tricarboxylic acid cycle | - | dehydrogenase | - |
| 30. | LEG1 | Galectin-1; LGALS1 | receptor binding | cellular process | - | signaling molecule; cell adhesion molecule | - |
| 31. | MOES | Moesin; MSN | structural constituent of cytoskeleton | cellular process; cellular component morphogenesis; cellular component organization | actin cytoskeleton; intracellular | actin family cytoskeletal protein | - |
| 32. | MUC18 | Cell surface glycoprotein MUC18; MCAM | receptor activity | cell communication; cell-cell adhesion; ectoderm development; nervous system development | - | receptor; immunoglobulin superfamily cell adhesion molecule | - |
| 33. | NEST | Nestin; NES | structural constituent of cytoskeleton | cellular process; cellular component morphogenesis; cellular component organization | intermediate filament cytoskeleton; intracellular | structural protein; intermediate filament | - |
| 34. | NQO1 | NAD(P)H dehydrogenase [quinone] 1; NQO1 | - | - | - | - | - |
| 35. | NSF1C | NSFL1 cofactor p47; NSFL1C | - | mitosis | - | membrane trafficking regulatory protein | - |
| 36. | PLST | Plastin-3; PLS3 | structural constituent of cytoskeleton; actin binding | cellular process; cellular component morphogenesis; cellular component organization | actin cytoskeleton; intracellular | non-motor actin binding protein | - |
| 37. | PPIA | Peptidyl-prolyl cis-trans isomerase A; PPIA | isomerase activity | immune system process; protein folding; intracellular protein transport; nuclear transport | - | isomerase | - |
| 38. | PRDX1 | Peroxiredoxin-1; PRDX1 | oxidoreductase activity; peroxidase activity | immune system process; metabolic process | - | peroxidase | - |
| 39. | PROF1 | Profilin-1; PFN1 | - | - | - | - | Cytoskeletal regulation by Rho GTPase->Profilin |
| 40. | PUR6 | Multifunctional protein ADE2; PAICS | ligase activity | purine nucleobase metabolic process | - | ligase | - |
| 41. | RAIN | Ras-interacting protein 1; RASIP1 | - | - | - | - | - |
| 42. | RAN | Ran-binding protein 6; RANBP6 | catalytic activity; transmembrane transporter activity; protein binding; small GTPase regulator activity | nucleobase-containing compound metabolic process; RNA localization; protein targeting; nuclear transport; regulation of catalytic activity | - | transporter; transfer/carrier protein; G-protein modulator | - |
| 43. | RAN | Ran-binding protein 17; RANBP17 | - | intracellular protein transport | - | transfer/carrier protein | - |
| 44. | RAN | Ran-binding protein 3; RANBP3 | catalytic activity; protein binding; small GTPase regulator activity | metabolic process; protein targeting; nuclear transport; regulation of catalytic activity | - | G-protein modulator | - |
| 45. | RAN | Ran GTPase-activating protein 1; RANGAP1 | - | - | - | - | - |
| 46. | RAN | GTP-binding nuclear protein Ran; RAN | GTPase activity; protein binding | nucleobase-containing compound metabolic process; cell cycle; cell communication; RNA localization; intracellular protein transport; nuclear transport | - | small GTPase | - |
| 47. | RAN | Ran-specific GTPase-activating protein; RANBP1 | catalytic activity; protein binding; small GTPase regulator activity | metabolic process; protein targeting; nuclear transport; regulation of catalytic activity | - | G-protein modulator | - |
| 48. | RAN | Ran guanine nucleotide release factor; RANGRF | - | - | - | - | - |
| 49. | RAN | Zinc finger Ran-binding domain-containing protein 2; ZRANB2 | - | - | - | - | - |
| 50. | RAN | Ran-binding protein 3-like; RANBP3L | catalytic activity; protein binding; small GTPase regulator activity | metabolic process; protein targeting; nuclear transport; regulation of catalytic activity | - | G-protein modulator | - |
| 51. | RAN | Ran-binding protein 10; RANBP10 | transmembrane transporter activity | intracellular protein transport; nuclear transport | - | transporter; transfer/carrier protein | - |
| 52. | RAN | Ran-binding protein 9; RANBP9 | transmembrane transporter activity | intracellular protein transport; nuclear transport | - | transporter; transfer/carrier protein | - |
| 53. | RCN1 | Reticulocalbin-1; RCN1 | calcium ion binding; calmodulin binding | cell communication | - | calmodulin | - |
| 54. | RL15 | 60S ribosomal protein L15; RPL15 | structural constituent of ribosome; nucleic acid binding | translation | - | ribosomal protein | - |
| 55. | RL19 | 60S ribosomal protein L19; RPL19 | structural constituent of ribosome; nucleic acid binding | translation | - | ribosomal protein | - |
| 56. | RS13 | 40S ribosomal protein S13; RPS13 | structural constituent of ribosome; nucleic acid binding | protein metabolic process | - | ribosomal protein | - |
| 57. | S10AD | Protein S100-A13; S100A13 | calcium ion binding; receptor binding; calmodulin binding | macrophage activation; DNA replication; cell cycle; cell communication | - | signaling molecule; calmodulin | - |
| 58. | SCRN1 | Secernin-1; SCRN1 | - | proteolysis | - | - | - |
| 59. | SDPR | Serum deprivation-response protein; SDPR | sequence-specific DNA binding transcription factor activity; sequence-specific DNA binding transcription factor activity | transcription from RNA polymerase II promoter; rRNA metabolic process | - | transcription factor | - |
| 60. | SH3L3 | SH3 domain-binding glutamic acid-rich-like protein 3; SH3BGRL3 | - | - | - | - | - |
| 61. | STIP1 | Stress-induced-phosphoprotein 1; STIP1 | - | protein folding; response to stress | - | chaperone | - |
| 62. | TAGL2 | Transgelin-2; TAGLN2 | structural constituent of cytoskeleton; actin binding | muscle contraction | actin cytoskeleton; intracellular | non-motor actin binding protein | - |
| 63. | TGM2 | Protein-glutamine gamma-glutamyltransferase 2; TGM2 | transferase activity, transferring acyl groups | cellular protein modification process | - | acyltransferase | - |
| 64. | THIO | Thioredoxin; TXN | oxidoreductase activity | respiratory electron transport chain; sulfur compound metabolic process; cell cycle; cell communication; response to stress | - | oxidoreductase | Hypoxia response via HIF activation->thioredoxin; Oxidative stress response->thioredoxin |
| 65. | TRXR1 | Thioredoxin reductase 1, cytoplasmic; TXNRD1 | oxidoreductase activity | immune system process; respiratory electron transport chain; nitrogen compound metabolic process; ferredoxin metabolic process | - | dehydrogenase; oxidase; reductase | - |
| 66. | TXND5 | Thioredoxin domain-containing protein 5; TXNDC5 | protein disulfide isomerase activity | protein folding; cellular protein modification process | - | isomerase | - |
| 67. | UB2L3 | Ubiquitin-conjugating enzyme E2 L3; UBE2L3 | ligase activity | apoptotic process; cellular protein modification process; apoptotic process | - | ligase | Ubiquitin proteasome pathway->Ubiquitin-conjugating enzyme E2; Parkinson disease->ubiquitin-conjugating enzyme E2L3 |
| 68. | UCHL1 | Ubiquitin carboxyl-terminal hydrolase isozyme L1; UCHL1 | cysteine-type peptidase activity | proteolysis | - | cysteine protease; cysteine protease | Parkinson disease->Ubiquitin C-terminal hydrolase-L1 |
| 69. | VAT1 | Synaptic vesicle membrane protein VAT-1 homolog; VAT1 | oxidoreductase activity | apoptotic process; carbohydrate metabolic process; apoptotic process | - | dehydrogenase; reductase | Huntington disease->Tumor protein p53 inducible protein 3 |
| 70. | VWF | von Willebrand factor; VWF | - | fertilization; cellular process; cell adhesion | extracellular region; extracellular matrix | extracellular matrix glycoprotein; cell adhesion molecule | Blood coagulation->von Willebrand Factor; Inflammation mediated by chemokine and cytokine signaling pathway->ExtraCellular matrix protein |
| 71. | WDR1 | WD repeat-containing protein 1; WDR1 | structural constituent of cytoskeleton; actin binding | cellular component movement | actin cytoskeleton; intracellular | non-motor actin binding protein | - |

Supplementary table 3. TC10

| Nr.crt. | Gene Symbol | Gene Name | Molecular Function | Biological Process | Cellular Component | Protein Class | Pathway |
| --- | --- | --- | --- | --- | --- | --- | --- |
| 1. | ACADV | Very long-chain specific acyl-CoA dehydrogenase, mitochondrial; ACADVL | oxidoreductase activity; transferase activity | respiratory electron transport chain; acyl-CoA metabolic process; nitrogen compound metabolic process; fatty acid beta-oxidation; acyl-CoA metabolic process | - | transferase; dehydrogenase; oxidase | - |
| 2. | AL1B1 | Aldehyde dehydrogenase X, mitochondrial; ALDH1B1 | oxidoreductase activity | nucleobase-containing compound metabolic process; cellular amino acid metabolic process | - | dehydrogenase | - |
| 3. | CO1A2 | Collagen alpha-2(I) chain; COL1A2 | receptor activity; extracellular matrix structural constituent; transmembrane transporter activity | immune system process; cell communication; cell-cell adhesion; cellular component morphogenesis; response to stimulus; intracellular protein transport; receptor-mediated endocytosis; regulation of liquid surface tension; cellular component organization | extracellular region; extracellular matrix | transporter; surfactant; receptor; extracellular matrix structural protein; antibacterial response protein | Integrin signalling pathway->Collagen |
| 4. | CP51A | Lanosterol 14-alpha demethylase; CYP51A1 | oxidoreductase activity | respiratory electron transport chain; lipid metabolic process | - | oxygenase | - |
| 5. | CYB5 | Cytochrome b5; CYB5A | oxidoreductase activity | respiratory electron transport chain; steroid metabolic process | - | oxidase | - |
| 6. | DHB12 | Estradiol 17-beta-dehydrogenase 12; HSD17B12 | oxidoreductase activity | cellular amino acid biosynthetic process; steroid metabolic process; visual perception; sensory perception | - | dehydrogenase; reductase | - |
| 7. | DHB4 | Peroxisomal multifunctional enzyme type 2; HSD17B4 | oxidoreductase activity | cellular amino acid biosynthetic process; steroid metabolic process | - | dehydrogenase; reductase | - |
| 8. | ECH1 | Delta(3,5)-Delta(2,4)-dienoyl-CoA isomerase, mitochondrial; ECH1 | oxidoreductase activity; acetyltransferase activity; hydro-lyase activity; racemase and epimerase activity; ligase activity | coenzyme metabolic process; vitamin biosynthetic process; carbohydrate metabolic process; fatty acid beta-oxidation | - | acetyltransferase; acyltransferase; dehydrogenase; hydratase; ligase; epimerase/racemase | - |
| 9. | ERGI1 | Endoplasmic reticulum-Golgi intermediate compartment protein 1; ERGIC1 | - | - | - | - | - |
| 10. | EZRI | Ezrin; EZR | structural constituent of cytoskeleton | cellular process; cellular component morphogenesis; cellular component organization | actin cytoskeleton; intracellular | actin family cytoskeletal protein | - |
| 11. | FINC | Fibronectin; FN1 | receptor binding | cell communication; cell-matrix adhesion; cell-cell adhesion | extracellular region | signaling molecule | Integrin signalling pathway->Fibronectin |
| 12. | K1C17 | Keratin, type I cytoskeletal 17; KRT17 | structural constituent of cytoskeleton | cellular process; cellular component morphogenesis; cellular component organization | intermediate filament cytoskeleton; intracellular | structural protein; intermediate filament | - |
| 13. | K1C18 | Keratin, type I cytoskeletal 18; KRT18 | structural constituent of cytoskeleton | cellular process; cellular component morphogenesis; cellular component organization | intermediate filament cytoskeleton; intracellular | structural protein; intermediate filament | - |
| 14. | K1C19 | Keratin, type I cytoskeletal 19; KRT19 | structural constituent of cytoskeleton | cellular process; cellular component morphogenesis; cellular component organization | intermediate filament cytoskeleton; intracellular | structural protein; intermediate filament | - |
| 15. | K2C6A | Keratin, type II cytoskeletal 6A; KRT6A | structural constituent of cytoskeleton | cellular process; cellular component morphogenesis; cellular component organization | intermediate filament cytoskeleton; intracellular | structural protein; intermediate filament | - |
| 16. | K2C7 | Keratin, type II cytoskeletal 7; KRT7 | structural constituent of cytoskeleton | cellular process; cellular component morphogenesis; cellular component organization | intermediate filament cytoskeleton; intracellular | structural protein; intermediate filament | - |
| 17. | K2C8 | Keratin, type II cytoskeletal 8; KRT8 | structural constituent of cytoskeleton | cellular process; cellular component morphogenesis; cellular component organization | intermediate filament cytoskeleton; intracellular | structural protein; intermediate filament | - |
| 18. | LPPRC | Leucine-rich PPR motif-containing protein, mitochondrial; LRPPRC | transmembrane receptor protein kinase activity; transmembrane receptor protein kinase activity; transmembrane transporter activity; RNA binding | protein phosphorylation; intracellular protein transport | - | transporter; serine/threonine protein kinase receptor; serine/threonine protein kinase receptor; RNA binding protein; protein kinase | - |
| 19. | MUC1 | Mucin-1; MUC1 | - | - | - | - | - |
| 20. | NB5R1 | NADH-cytochrome b5 reductase 1; CYB5R1 | oxidoreductase activity | respiratory electron transport chain; nitrogen compound metabolic process; unsaturated fatty acid biosynthetic process; cholesterol metabolic process | - | reductase | - |
| 21. | NOP2 | Putative ribosomal RNA methyltransferase NOP2; NOP2 | - | - | - | - | - |
| 22. | OAT | Ornithine aminotransferase, mitochondrial; OAT | transaminase activity | vitamin biosynthetic process; cellular amino acid biosynthetic process | - | transaminase | - |
| 23. | PGRC1 | Membrane-associated progesterone receptor component 1; PGRMC1 | receptor activity; receptor binding | cell communication | - | signaling molecule; receptor | - |
| 24. | PLOD2 | Procollagen-lysine,2-oxoglutarate 5-dioxygenase 2; PLOD2 | oxidoreductase activity | protein metabolic process; cellular process; cell adhesion | - | oxygenase | - |
| 25. | PTGIS | Prostacyclin synthase; PTGIS | oxidoreductase activity; isomerase activity | respiratory electron transport chain; fatty acid biosynthetic process; blood circulation; regulation of vasoconstriction | - | oxidoreductase; isomerase | - |
| 26. | RRS1 | Ribosome biogenesis regulatory protein homolog; RRS1 | structural constituent of ribosome; nucleic acid binding | nucleobase-containing compound metabolic process | - | ribosomal protein | - |
| 27. | SERA | D-3-phosphoglycerate dehydrogenase; PHGDH | oxidoreductase activity | carbohydrate metabolic process; cellular amino acid biosynthetic process | - | dehydrogenase | Serine glycine biosynthesis->Phosphoglycerate dehydrogenase |
| 28. | SODM | Superoxide dismutase [Mn], mitochondrial; SOD2 | oxidoreductase activity | metabolic process | - | oxidoreductase | - |
| 29. | SQRD | Sulfide:quinone oxidoreductase, mitochondrial; SQRDL | - | - | - | - | - |
| 30. | TAGL | Transgelin; TAGLN | structural constituent of cytoskeleton; actin binding | muscle contraction | actin cytoskeleton; intracellular | non-motor actin binding protein | - |
| 31. | THIM | 3-ketoacyl-CoA thiolase, mitochondrial; ACAA2 | acetyltransferase activity | protein acetylation | - | acetyltransferase | - |

Supplementary table 4. EC10

| Nr.crt. | Gene Symbol | Gene Name | Molecular Function | Biological Process | Cellular Component | Protein Class | Pathway |
| --- | --- | --- | --- | --- | --- | --- | --- |
| 1. | 6PGD | 6-phosphogluconate dehydrogenase, decarboxylating; PGD | oxidoreductase activity | pentose-phosphate shunt | - | dehydrogenase | Pentose phosphate pathway->Gluconate Dehydrogenase |
| 2. | ACTN1 | Alpha-actinin-1; ACTN1 | structural constituent of cytoskeleton; actin binding | cellular component movement; cellular component morphogenesis; cellular component organization | actin cytoskeleton; intracellular | non-motor actin binding protein | Integrin signalling pathway->alpha actinin |
| 3. | ACTN4 | Alpha-actinin-4; ACTN4 | structural constituent of cytoskeleton; actin binding | cellular component movement; cellular component morphogenesis; cellular component organization | actin cytoskeleton; intracellular | non-motor actin binding protein | Integrin signalling pathway->alpha actinin |
| 4. | ALDOA | Fructose-bisphosphate aldolase A; ALDOA | - | - | - | - | Fructose galactose metabolism->Fructose bisphosphate aldolase; Glycolysis->Aldolase |
| 5. | AMPN | Aminopeptidase N; ANPEP | metallopeptidase activity | proteolysis | - | metalloprotease; metalloprotease | - |
| 6. | CATB | Cathepsin B; CTSB | cysteine-type peptidase activity | proteolysis | - | cysteine protease; cysteine protease | - |
| 7. | CNN2 | Calponin-2; CNN2 | structural constituent of cytoskeleton; actin binding | muscle contraction | actin cytoskeleton; intracellular | non-motor actin binding protein | - |
| 8. | COTL1 | Coactosin-like protein; COTL1 | structural constituent of cytoskeleton; actin binding | cellular process; cellular component organization | actin cytoskeleton; intracellular | non-motor actin binding protein | - |
| 9. | CRIP2 | Cysteine-rich protein 2; CRIP2 | sequence-specific DNA binding transcription factor activity; structural constituent of cytoskeleton; sequence-specific DNA binding transcription factor activity; RNA binding | gamete generation; immune system process; apoptotic process; transcription from RNA polymerase II promoter; cellular component movement; ectoderm development; mesoderm development; cellular component morphogenesis; embryo development; apoptotic process; nervous system development; muscle organ development; cellular defense response; regulation of transcription from RNA polymerase II promoter; negative regulation of apoptotic process; cellular component organization | actin cytoskeleton; intracellular | homeobox transcription factor; zinc finger transcription factor; structural protein; RNA binding protein; actin family cytoskeletal protein | - |
| 10. | CSRP1 | Cysteine and glycine-rich protein 1; CSRP1 | structural constituent of cytoskeleton | immune system process; muscle organ development | actin cytoskeleton; intracellular | actin family cytoskeletal protein | - |
| 11. | DEST | Destrin; DSTN | structural constituent of cytoskeleton; actin binding | protein complex assembly; cellular process; cellular component morphogenesis; cellular component organization; protein complex biogenesis | actin cytoskeleton; intracellular | non-motor actin binding protein | - |
| 12. | DPYL2 | Dihydropyrimidinase-related protein 2; DPYSL2 | hydrolase activity | nucleobase-containing compound metabolic process | - | hydrolase | Axon guidance mediated by semaphorins->Collapsin response mediator protein; Pyrimidine Metabolism->Dihydropyrimidinase |
| 13. | EGLN | Endoglin; ENG | transforming growth factor beta-activated receptor activity; transforming growth factor beta-activated receptor activity; cytokine receptor activity; transforming growth factor beta-activated receptor activity | protein phosphorylation; cell communication; mesoderm development; angiogenesis; heart development | - | TGF-beta receptor | - |
| 14. | EHD2 | EH domain-containing protein 2; EHD2 | catalytic activity; calcium ion binding; protein binding; small GTPase regulator activity | metabolic process; synaptic transmission; neurotransmitter secretion; intracellular protein transport; endocytosis; regulation of catalytic activity | - | membrane traffic protein; G-protein modulator; calcium-binding protein | - |
| 15. | ENOA | Alpha-enolase; ENO1 | lyase activity | glycolysis; glycolysis | - | lyase | Glycolysis->Enolase |
| 16. | FABP5 | Fatty acid-binding protein, epidermal; FABP5 | lipid binding | lipid metabolic process; cell communication; ectoderm development; lipid transport; vitamin transport | - | transfer/carrier protein | - |
| 17. | FETUA | Alpha-2-HS-glycoprotein; AHSG | cysteine-type peptidase activity; protein binding; cysteine-type endopeptidase inhibitor activity | immune system process; proteolysis; mesoderm development; skeletal system development; regulation of catalytic activity | extracellular region; extracellular matrix | extracellular matrix glycoprotein; cysteine protease inhibitor | - |
| 18. | FKB1A | Peptidyl-prolyl cis-trans isomerase FKBP1A; FKBP1A | isomerase activity; binding | cellular protein modification process; cellular process | cytoplasm; neuron projection | isomerase; chaperone; calcium-binding protein | TGF-beta signaling pathway->FK506 binding protein 12KDa |
| 19. | FLNB | Filamin-B; FLNB | structural constituent of cytoskeleton; actin binding | cellular component movement; cellular component morphogenesis; cellular component organization | actin cytoskeleton; intracellular | non-motor actin binding protein | Integrin signalling pathway->Filamin |
| 20. | FSCN1 | Fascin; FSCN1 | structural constituent of cytoskeleton; actin binding | cellular component movement | actin cytoskeleton; intracellular | non-motor actin binding protein | - |
| 21. | GPX1 | Glutathione peroxidase 1; GPX1 | oxidoreductase activity; peroxidase activity | immune system process; steroid metabolic process; response to stress; response to toxic substance | - | peroxidase | - |
| 22. | H12 | Histone H1.2; HIST1H1C | DNA binding | nucleobase-containing compound metabolic process; cellular process; chromatin organization | - | histone | - |
| 23. | H15 | Histone H1.5; HIST1H1B | DNA binding | nucleobase-containing compound metabolic process; cellular process; chromatin organization | - | histone | - |
| 24. | HSPB1 | Heat shock protein beta-1; HSPB1 | structural molecule activity | immune system process; protein folding; muscle contraction; visual perception; sensory perception; response to stress | - | structural protein; chaperone | p38 MAPK pathway->heat shock protein 27kD; Angiogenesis->Heat-Shock Protein 27; VEGF signaling pathway->Heat-Shock Protein 27 |
| 25. | HSPB1 | HSPB1-associated protein 1; HSPBAP1 | - | - | - | - | - |
| 26. | ITA5 | Integrin alpha-5; ITGA5 | - | cellular process; cell adhesion | - | cell adhesion molecule | Integrin signalling pathway->Integrin alpha |
| 27. | K6PP | 6-phosphofructokinase type C; PFKP | carbohydrate kinase activity | glycolysis; glycolysis | - | carbohydrate kinase; carbohydrate kinase | - |
| 28. | MARE1 | Microtubule-associated protein RP/EB family member 1; MAPRE1 | structural constituent of cytoskeleton; microtubule binding | cell cycle; cellular component morphogenesis; cellular component organization | microtubule; cytoskeleton; intracellular | non-motor microtubule binding protein | - |
| 29. | MOES | Moesin; MSN | structural constituent of cytoskeleton | cellular process; cellular component morphogenesis; cellular component organization | actin cytoskeleton; intracellular | actin family cytoskeletal protein | - |
| 30. | MUC18 | Cell surface glycoprotein MUC18; MCAM | receptor activity | cell communication; cell-cell adhesion; ectoderm development; nervous system development | - | receptor; immunoglobulin superfamily cell adhesion molecule | - |
| 31. | NEST | Nestin; NES | structural constituent of cytoskeleton | cellular process; cellular component morphogenesis; cellular component organization | intermediate filament cytoskeleton; intracellular | structural protein; intermediate filament | - |
| 32. | NTF2 | Nuclear transport factor 2; NUTF2 | - | intracellular protein transport; nuclear transport | - | - | - |
| 33. | NTF2 | NTF2-related export protein 2; NXT2 | - | intracellular protein transport; nuclear transport | - | - | - |
| 34. | NTF2 | NTF2-related export protein 1; NXT1 | - | intracellular protein transport; nuclear transport | - | - | - |
| 35. | P3H3 | Prolyl 3-hydroxylase 3; LEPREL2 | - | cell communication; cell-cell adhesion | extracellular region; extracellular matrix | extracellular matrix glycoprotein | - |
| 36. | PDLI1 | PDZ and LIM domain protein 1; PDLIM1 | sequence-specific DNA binding transcription factor activity; structural constituent of cytoskeleton; sequence-specific DNA binding transcription factor activity; actin binding | transcription from RNA polymerase II promoter; cellular component movement; muscle contraction; mesoderm development; cellular component morphogenesis; heart development; muscle organ development; regulation of transcription from RNA polymerase II promoter; cellular component organization | actin cytoskeleton; intracellular | transcription factor; non-motor actin binding protein | - |
| 37. | PDLI7 | PDZ and LIM domain protein 7; PDLIM7 | sequence-specific DNA binding transcription factor activity; structural constituent of cytoskeleton; sequence-specific DNA binding transcription factor activity; actin binding | transcription from RNA polymerase II promoter; cellular component movement; muscle contraction; mesoderm development; cellular component morphogenesis; skeletal system development; heart development; muscle organ development; regulation of transcription from RNA polymerase II promoter; cellular component organization | actin cytoskeleton; intracellular | transcription factor; non-motor actin binding protein | - |
| 38. | PEA15 | Astrocytic phosphoprotein PEA-15; PEA15 | - | - | - | - | Gonadotropin releasing hormone receptor pathway->PEA-15 |
| 39. | PLOD1 | Procollagen-lysine,2-oxoglutarate 5-dioxygenase 1; PLOD1 | oxidoreductase activity | protein metabolic process; cellular process; cell adhesion | - | oxygenase | - |
| 40. | PLST | Plastin-3; PLS3 | structural constituent of cytoskeleton; actin binding | cellular process; cellular component morphogenesis; cellular component organization | actin cytoskeleton; intracellular | non-motor actin binding protein | - |
| 41. | PROF1 | Profilin-1; PFN1 | - | - | - | - | Cytoskeletal regulation by Rho GTPase->Profilin |
| 42. | PTRF | Polymerase I and transcript release factor; PTRF | sequence-specific DNA binding transcription factor activity; sequence-specific DNA binding transcription factor activity | transcription from RNA polymerase II promoter; rRNA metabolic process | - | transcription factor | General transcription by RNA polymerase I->PTRF |
| 43. | RL24 | 60S ribosomal protein L24; RPL24 | structural constituent of ribosome; nucleic acid binding | translation | - | ribosomal protein | - |
| 44. | RL7A | 60S ribosomal protein L7a; RPL7A | structural constituent of ribosome; nucleic acid binding | rRNA metabolic process; translation | - | ribosomal protein | - |
| 45. | RL7A | 60S ribosomal protein L7a; RPL7A | - | - | - | - | - |
| 46. | SDPR | Serum deprivation-response protein; SDPR | sequence-specific DNA binding transcription factor activity; sequence-specific DNA binding transcription factor activity | transcription from RNA polymerase II promoter; rRNA metabolic process | - | transcription factor | - |
| 47. | SH3L3 | SH3 domain-binding glutamic acid-rich-like protein 3; SH3BGRL3 | - | - | - | - | - |
| 48. | SPHM | N-sulphoglucosamine sulphohydrolase; SGSH | hydrolase activity | sulfur compound metabolic process; phospholipid metabolic process; polysaccharide metabolic process; phospholipid metabolic process | - | hydrolase | - |
| 49. | SYWC | Tryptophan--tRNA ligase, cytoplasmic; WARS | aminoacyl-tRNA ligase activity | translation | cytoplasm | aminoacyl-tRNA synthetase | - |
| 50. | TGM2 | Protein-glutamine gamma-glutamyltransferase 2; TGM2 | transferase activity, transferring acyl groups | cellular protein modification process | - | acyltransferase | - |
| 51. | TPIS | Triosephosphate isomerase; TPI1 | isomerase activity | glycolysis; glycolysis | - | isomerase | Glycolysis->Triosephosphate isomerase |
| 52. | TPP1 | Tripeptidyl-peptidase 1; TPP1 | serine-type peptidase activity | proteolysis | - | serine protease; serine protease | - |
| 53. | TSP1 | Thrombospondin-1; THBS1 | - | - | - | - | p53 pathway->Thrombospondin-1 |
| 54. | TXND5 | Thioredoxin domain-containing protein 5; TXNDC5 | protein disulfide isomerase activity | protein folding; cellular protein modification process | - | isomerase | - |
| 55. | VAT1 | Synaptic vesicle membrane protein VAT-1 homolog; VAT1 | oxidoreductase activity | apoptotic process; carbohydrate metabolic process; apoptotic process | - | dehydrogenase; reductase | Huntington disease->Tumor protein p53 inducible protein 3 |
| 56. | VIME | Vimentin; VIM | structural constituent of cytoskeleton | cellular process; cellular component morphogenesis; cellular component organization | intermediate filament cytoskeleton; intracellular | structural protein; intermediate filament | - |
| 57. | VWF | von Willebrand factor; VWF | - | fertilization; cellular process; cell adhesion | extracellular region; extracellular matrix | extracellular matrix glycoprotein; cell adhesion molecule | Blood coagulation->von Willebrand Factor; Inflammation mediated by chemokine and cytokine signaling pathway->ExtraCellular matrix protein |
| 58. | WDR1 | WD repeat-containing protein 1; WDR1 | structural constituent of cytoskeleton; actin binding | cellular component movement | actin cytoskeleton; intracellular | non-motor actin binding protein | - |
| 59. | ZYX | Zyxin; ZYX | sequence-specific DNA binding transcription factor activity; kinase activity; structural constituent of cytoskeleton; sequence-specific DNA binding transcription factor activity; protein binding; kinase regulator activity | transcription from RNA polymerase II promoter; cellular component movement; mitosis; sensory perception; ectoderm development; cellular component morphogenesis; nervous system development; regulation of transcription from RNA polymerase II promoter; regulation of catalytic activity; cellular component organization | plasma membrane; cell junction; actin cytoskeleton; intracellular | homeobox transcription factor; zinc finger transcription factor; nucleic acid binding; kinase modulator; actin family cytoskeletal protein; cell junction protein | - |
